# Offspring susceptibility to metabolic alterations due to maternal high-fat diet and the impact of inhaled ozone used as a stressor

**DOI:** 10.1038/s41598-020-73361-0

**Published:** 2020-10-01

**Authors:** Samantha J. Snow, Katarzyna Broniowska, Edward D. Karoly, Andres R. Henriquez, Pamela M. Phillips, Allen D. Ledbetter, Mette C. Schladweiler, Colette N. Miller, Christopher J. Gordon, Urmila P. Kodavanti

**Affiliations:** 1 Public Health and Integrated Toxicology Division, Center for Public Health and Environmental Assessment, US Environmental Protection Agency, 109 T.W. Alexander Dr. Research Triangle Park, Durham, NC USA; 2 Metabolon Inc, Durham, NC USA; 3 Oak Ridge Institute for Science and Education, Durham, NC USA; 4 Present Address: ICF Inc, Durham, NC USA

**Keywords:** Developmental biology, Diseases

## Abstract

The influence of maternal high-fat diet (HFD) on metabolic response to ozone was examined in Long-Evans rat offspring. F0 females were fed control diet (CD; 10%kcal from fat) or HFD (60%kcal from fat) starting at post-natal day (PND) 30. Rats were bred on PND 72. Dietary regimen was maintained until PND 30 when all offspring were switched to CD. On PND 40, F1 offspring (n = 10/group/sex) were exposed to air or 0.8 ppm ozone for 5 h. Serum samples were collected for global metabolomic analysis (n = 8/group/sex). Offspring from HFD dams had increased body fat and weight relative to CD. Metabolomic analysis revealed significant sex-, diet-, and exposure-related changes. Maternal HFD increased free fatty acids and decreased phospholipids (male > female) in air-exposed rats. Microbiome-associated histidine and tyrosine metabolites were increased in both sexes, while 1,5-anhydroglucitol levels decreased in males indicating susceptibility to insulin resistance. Ozone decreased monohydroxy fatty acids and acyl carnitines and increased pyruvate along with TCA cycle intermediates in females (HFD > CD). Ozone increased various amino acids, polyamines, and metabolites of gut microbiota in HFD female offspring indicating gut microbiome alterations. Collectively, these data suggest that maternal HFD increases offspring susceptibility to metabolic alterations in a sex-specific manner when challenged with environmental stressors.

## Introduction

The incidence of metabolic disorders has increased worldwide. The US Center for Disease Control 2015–2016 report indicates that 38.9% of US adults and youth are obese^1^. In 2015, an estimated 30.3 million people of all ages (9.4% of the US population) had diabetes; this was most prevalent in American Indians/Alaska Natives, non-Hispanic blacks, and people of Hispanic ethnicity than among non-Hispanic whites (https://www.cdc.gov/diabetes/pdfs/data/statistics/national-diabetes-statistics-report.pdf). Several lifestyle risk factors have been implicated in the increased prevalence of metabolic disorders, such as calorie-rich diets, large portion sizes, low activity levels, and increased psychosocial stresses^2,3^. Environmental exposures have also been predicted to increase the incidence of metabolic disease. Exposure to ambient particulate matter > 2.5 µm in diameter contributed to about 3.2 million incident cases of diabetes globally^4^. Moreover, the link between air pollution and obesity may be influenced by physiological and social factors^5^.

Outdoor air pollution accounts for nearly 7 million premature deaths worldwide annually based on the World Health Organization report of May 2018 and nearly 70% of deaths attributed to environmental causes are due to air pollution^6^. Tropospheric ozone, one of the ubiquitous criteria air pollutants, is generated by interaction with anthropogenic emissions and sunlight, and its levels are expected to increase due to climate change^7^. Ozone has been extensively studied for its pulmonary effects; however, more recently neurological and systemic effects have also been reported^8^. We have shown that similar to many non-chemical (psychosocial) stressors^9^, a single acute ozone inhalation produces a myriad of changes in metabolic and immunological processes through sympathetic and hypothalamic neuroendocrine stress pathway activation^10,11^. Thus, acute ozone exposure as a stressor may alter a broad spectrum of homeostatic processes, which in susceptible individuals may contribute to exacerbation of metabolic disease.

Maternal obesity and calorie-rich diets have been implicated in offspring predisposition to metabolic diseases through the developmental reprogramming of metabolic regulation in the hypothalamus^12^. Sex-dependent effects of maternal obesity are linked to impairment of insulin, glucose, and lipid metabolism in multiple tissues of rat offspring^13^. It is postulated that circulating lipid metabolites from obese mothers likely influence the fetal metabolic phenotype during development^14^. Transcriptomic assessment of baboon fetal livers born to mothers fed high-fat/high-fructose diet indicated dysregulation of the TCA cycle, glycolysis, changes in Wnt/β-catenin signaling, and marked lipid accumulation^15^. Epigenome-wide methylation changes^16^ and hypothalamic leptin and insulin resistance are postulated to be contributing factors^17^. These studies provide evidence that maternal obesity is a risk factor for offspring metabolic programming.

Maternal exposure to endocrine disrupting chemicals, psychosocial stressors, and steroids during pregnancy has also been postulated to interactively alter fetal hypothalamic programming of metabolic homeostasis and increase the offspring’s susceptibility to metabolic diseases^18^. A number of mechanisms have been proposed including early alterations of fetal metabolic programming in the hypothalamus, influence of placental adaptation, changes in epigenome, and alterations of gut microbiome^19^. Although experimental studies have examined how maternal exposure to environmental chemicals increases the risk of diabetes and obesity in offspring^20,21^, no prior studies have examined how offspring born to obese mothers fed a high-fat diet (HFD) may respond differently to an acute exposure of an inhaled environmental pollutant. We postulated that maternal HFD increases the risk of offspring to develop systemic metabolic alterations. We further proposed that these metabolic alterations in the offspring induced by maternal high fat diet will be exacerbated after exposure to an environmental stressor, ozone. This was based on our recent findings that exposure to air pollutants, such as ozone and acrolein, induces a wide array of systemic metabolic changes in rodents^22–26^ and in humans^27^ through the activation of neuroendocrine stress pathways involving the sympathetic–adrenal–medullary and hypothalamic–pituitary–adrenal axes^10,28^. We hypothesized that offspring born to obese mothers on HFD when challenged with such an environmental stressor, will produce exacerbated metabolic alterations which will unmask the susceptibility in offspring to metabolic impairment and reveal maternal contribution to childhood metabolic disease. In this study, we examined if maternal obesity and HFD altered ozone-induced hormonal and metabolic changes in peri-adolescent male and female offspring using clinical and hormonal assessments, and global serum metabolomics. The susceptibility of maternal HFD and obesity on pulmonary injury and inflammation following post-natal exposure to ozone was also determined.

## Results

### Body weights and body fat composition of dams and offspring

**Figure 1:**
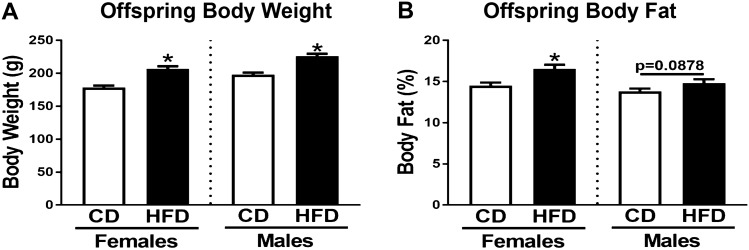
Offspring body weight and body fat % are increased by maternal high-fat diet (HFD). Body weight (**A**) and body fat % (**B**) were assessed at PND 37 for female and male offspring of dams fed a control diet (CD) or HFD. Values represent mean ± SEM, n = 20 offspring (2 offspring/dam/sex). *Significant effect of maternal diet (*p* < 0.05).

We have established a model of maternal diet-induced obesity by feeding 60% HFD to female LE rats starting from PND 30^29^. As reported earlier, females on HFD were heavier and had greater body fat % when compared to those on CD during gestation^30^. Forty-day-old female and male offspring from HFD dams were also significantly heavier than those from CD dams (Fig. 1A). The body fat % of the female offspring from HFD dams likewise was significantly greater relative to offspring from CD dams; this difference was smaller for male offspring (*p* = 0.088) (Fig. 1B).

### Effects of maternal HFD on ozone-induced lung injury/inflammation, and circulating hormones and lipids

Ozone exposure has been shown to induce lung injury and inflammation in many of our rat studies^25,31^, which begins to occur immediately-post exposure but peaks next day^23,31^. To determine lung injury in male and female offspring immediately after a single 5 h ozone exposure, we assessed BALF injury markers and neutrophilic inflammation. Ozone exposure increased markers of pulmonary protein leakage and lung cell injury (i.e., protein, albumin, and NAG activity) similarly in both sexes and dietary groups. However, neutrophilic inflammation was more evident after ozone exposure in female offspring. Neutrophilic inflammation was minimal at this early time point in male offspring (Supplementary Fig. S1).

Since ozone-induced metabolic effects have been shown to be mediated through the activation of neuroendocrine stress pathways in the male Wistar–Kyoto rat strain^23,24^, we sought to determine if young LE offspring would demonstrate similar effects and whether maternal diet modified this response. Maternal HFD decreased FSH in male air- and ozone-exposed offspring but had no effect on other hormones (Supplementary Fig. S2). Ozone exposure decreased ACTH in female offspring from CD dams and increased PRL in female offspring from HFD dams (Supplementary Fig. S2). In male offspring, no significant ozone-induced changes were noted regardless of maternal diet (Supplemental Materials, Fig. 2).

**Figure 2:**
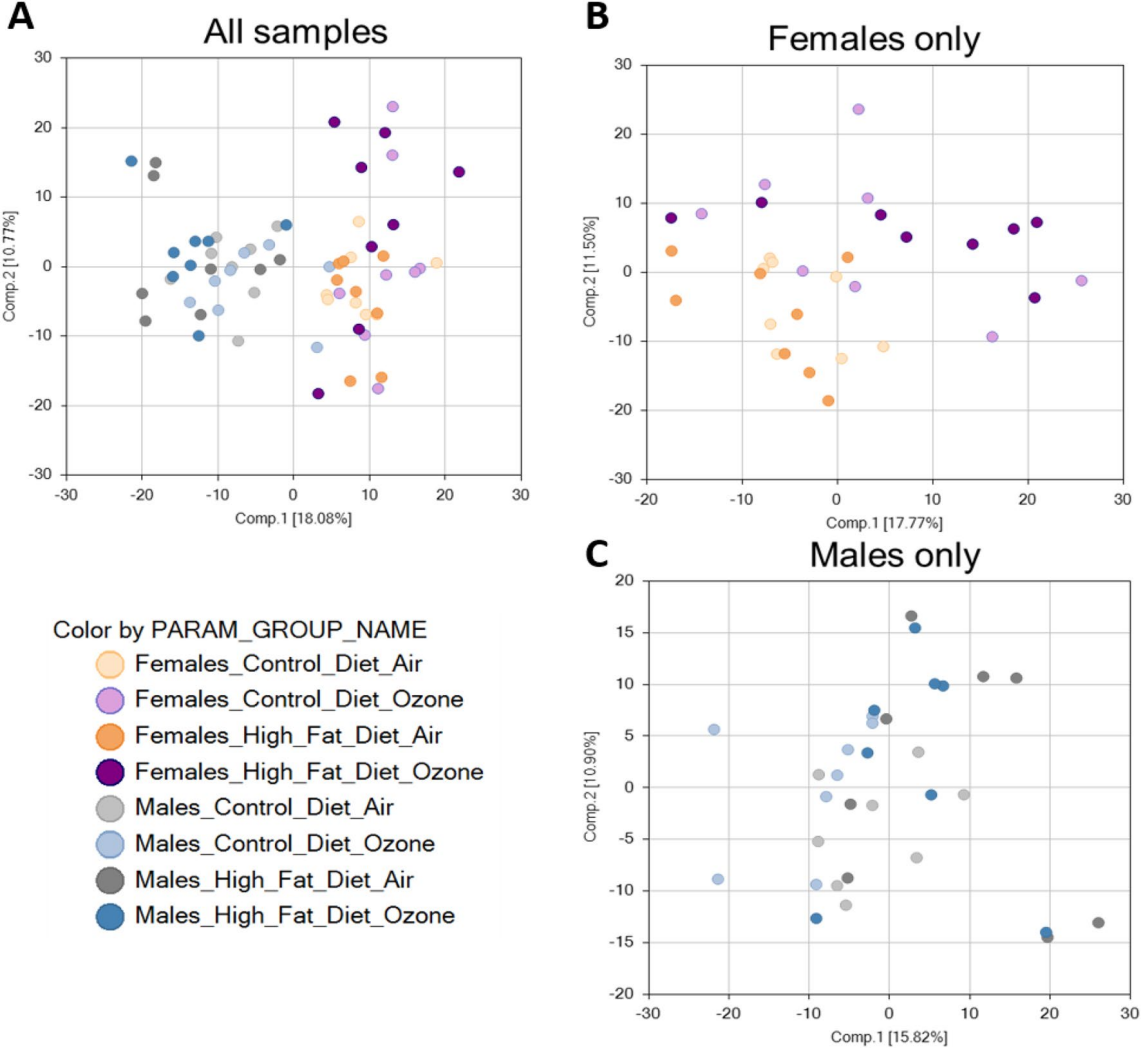
Principal component analysis depicting changes in serum metabolites for female and male offspring born to mothers fed control diet (CD) or high-fat diet (HFD) and exposed to air or ozone. (**A**; all 64 samples, n = 8/group), females only (**B**; 32 samples, n = 8/group), and males only (**C**; 32 samples, n = 8/group). Female and male offspring from dams fed control diet or high-fat diet were exposed to air or ozone (5 h) and serum samples were assessed using global metabolomics.

No significant maternal diet-related or acute ozone exposure-induced changes were noted in levels of circulating stress hormones (i.e., corticosterone and adrenaline) or metabolic hormones (i.e., insulin and leptin) in male or female offspring (Supplementary Figs. S3, S4, respectively). Circulating total triglycerides and FFA were also not affected by maternal diet or ozone exposure in male or female offspring. However, total cholesterol was lower in ozone-exposed female offspring from HFD dams as well as in air- and ozone-exposed male offspring from HFD dams compared to the offspring from CD dams. Maternal HFD effect was also reflected in lower levels of LDL and HDL in air-exposed male offspring (Supplementary Fig. S5).

### Serum metabolomics

A total of 737 compounds were identified through metabolomic assessment. A summary of the numbers of biochemicals that achieved statistical significance (*p* ≤ 0.05), as well as those approaching significance (0.05 < *p* < 0.10), is shown in Table 1. Analysis by three-way ANOVA identified biochemicals exhibiting significant interaction and main effects for experimental parameters of exposure, diet, and sex. Interestingly, ozone-exposed females regardless of maternal diet were characterized by higher intra-group variability than the remaining groups as evidenced by principal component analysis (PCA; Fig. 2). Additional PCA for female and male rats separately showed that in the case of females, ozone exposure resulted in segregation along component 2, whereas no additional separation was observed for male rats (Fig. 2). These results correspond with the number of statistically significant differences detected in the dataset (Table 1). Ninety-five metabolites were significantly affected by ozone in males from CD and HFD dams whereas 153 metabolites were affected by ozone in females from CD dams. This number was further increased to 190 metabolites affected by ozone exposure in females from HFD dams. Both PCA results and the number of statistically different metabolites in the current dataset suggest that ozone exposure elicits greater metabolic impact in female than male rats, and that maternal HFD may further potentiate this effect in female offspring.

**Table 1:** Significant changes in the number of circulating metabolites depicting the influence of maternal high-fat diet (HFD) on male and female offspring exposed to air or ozone.

	Exposure effect			
	CD ozone F CD air F	HFD ozone F HFD air F	CD ozone M CD air M	HFD ozone M HFD air M
Total biochemicals (*p* ≤ 0.05)	153	190	95	95
Biochemicals (↑ | ↓)	88 | 65	114 | 76	62 | 33	50 | 45
Total biochemicals (0.05 < *p* < 0.10)	57	62	50	51
Biochemicals (↑ | ↓)	31 | 26	45 | 17	27 | 23	34 | 17

	Diet effect			
	HFD air F CD air F	HFD ozone F CD ozone F	HFD air M CD air M	HFD ozone M CD ozone M
Total biochemicals (*p* ≤ 0.05)	48	57	88	116
Biochemicals (↑ | ↓)	19 | 29	31 | 26	46 | 42	51 | 65
Total biochemicals (0.05 < *p* < 0.10)	40	47	53	51
Biochemicals (↑ | ↓)	18 | 22	26 | 21	30 | 23	20 | 31

	Sex effect			
	CD air M CD air F	HFD air M HFD air F	CD ozone M CD ozone F	HFD ozone M HFD ozone F
Total biochemicals (*p* ≤ 0.05)	220	260	232	307
Biochemicals (↑ | ↓)	149 | 71	178 | 82	172 | 60	193 | 114
Total biochemicals (0.05 < *p* < 0.10)	54	44	57	43
Biochemicals (↑ | ↓)	33 | 21	25 | 19	42 | 15	26 | 17

Number of metabolites significantly changed in serum (at *p* ≤ 0.05 and 0.05 < *p* < 0.10) by exposure, diet, or sex in young male and female offspring (n = 8/group) from dams that were fed control diet (CD) or HFD and exposed to air or 0.8 ppm ozone for 5 h. Data were analyzed using three-way analysis of variance. ↑ indicates increase and ↓ indicates decrease in number of biochemicals significantly changed.

## Effect of maternal HFD on serum metabolites for air-exposed male and female offspring

Changes in the profile of circulating metabolites due to maternal HFD can inform how the offspring may have impaired metabolic regulation, and how they will respond to ozone, a challenge stressor, to mediate metabolic homeostatic changes. Specific maternal diet-induced metabolite differences in major metabolic processes are shown in Supplementary Fig. S6 and are summarized in Table 2. These data demonstrate that maternal HFD decreases circulating anhydroglucitol in air-exposed male offspring, which may be linked to insulin resistance (Supplementary Fig. S6). Maternal diet-related increases in long-chain and poly-unsaturated fatty acids along with alterations in various carnitines were noted in both male and female offspring. Maternal HFD-induced changes in lysophospholipids, monoacylglycerols, and diacylglycerols were noted primarily in males, whereas ceramides were increased in female offspring (Supplementary Fig. S6). Benzoate metabolites were decreased in air-exposed male and female offspring while phenol sulfate and phenol glucuronide were increased in female offspring from HFD dams, suggesting changes in gut microbiome (Table 2; Supplementary Fig. S6).

**Table 2:** Summary of maternal high-fat diet (HFD)-induced changes in serum metabolites in 40-day-old male and female offspring exposed to air.

Female diet effect	Male diet effect	Interpretation
**Microbiome**		
↑ in microbial catabolites of tyrosine—phenol sulfate and phenol glucuronide (HFD > CD) ↓ in benzoate metabolites	↓ in benzoate metabolites ↑ in 3-(3-hydroxyphenyl) propionate sulfate	Maternal HFD could change male and female offspring microbiome that might be linked to metabolic diseases
**Energy metabolism**		
→ in anhydroglucitol (1,5-AG)	↓ Anhydroglucitol (1,5-AG)	Likely increased competition with glucose for renal clearance Susceptibility to insulin resistance
**Lipid metabolism**		
↑ and ↓ in BCAA metabolites ↑ in polyunsaturated fatty acids and long chain fatty acids ↓ in medium chain fatty acids	↑ in polyunsaturated fatty acids ↓ in medium and short chain fatty acids, lysophospholipids, and monoacyl glycerols	Maternal HFD induced changes in circulating lipids (males > females). These changes may be secondary to altered liver metabolic processes
↑ in ceramides ↓ in sterol metabolites	↑ in pyrimidine metabolites ↓ in sterol metabolites	Sterol metabolism inhibited in both sexes while sex-specific effects on ceramides may indicate liver susceptibility to steatosis-like changes

Data are summarized from the metabolomics report on diet-induced changes in serum metabolites in young male and female offspring (n = 8/group) exposed to air from dams that were fed CD or HFD. ↑ indicates increase in metabolite levels, ↓ indicates decrease in metabolite levels, and → indicates no change in metabolite levels.

*BCAA* branched-chain amino acids.

## Effect of ozone on serum metabolites in male and female offspring from CD and HFD dams

### Changes in lipid metabolism

**Figure 3:**
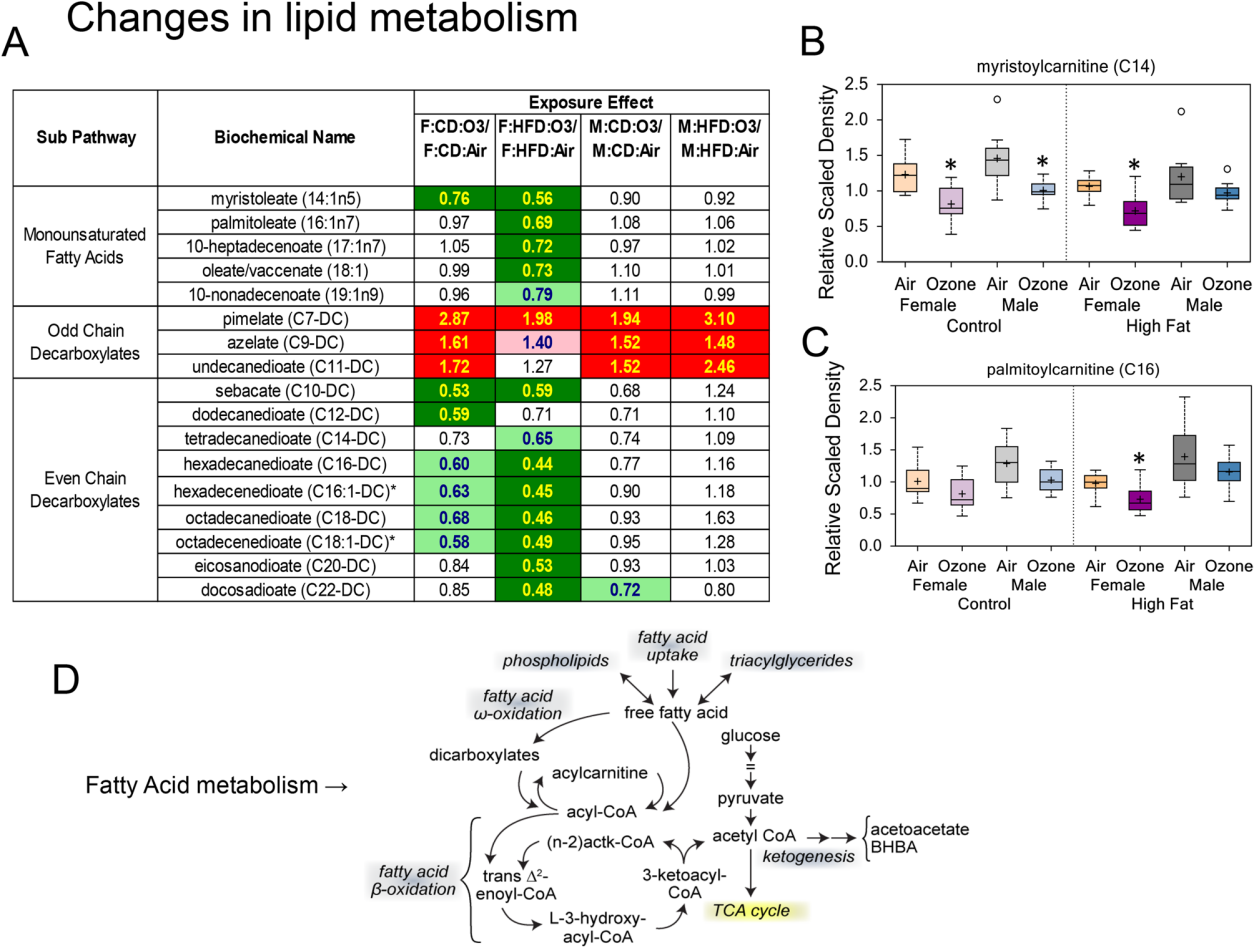
Alterations in serum lipid metabolites in young female and male offspring from dams that were fed control diet (CD) or high-fat diet (HFD): the influence of acute ozone exposure. Relative fold differences between ozone- and air-exposed female and male offspring (n = 8/group) (**A**). The values show mean fold change due to ozone exposure when compared to filtered air. Red or green indicates p ≤ 0.05; pink or light green indicates 0.05 < p < 0.10. Box plots demonstrating ozone-induced changes in myristoylcarnitine [C14] (**B**), and palmitoylcarnitine [C16] (**C**). *Significant effect of ozone exposure (p < 0.10 or p < 0.05) when compared to matching air group. A schema highlighting fatty acid metabolism pathways impacted by ozone exposure (**D**).

There were many ozone exposure-related changes observed in lipid metabolites in male and female offspring from CD and HFD dams (Table 3; Supplementary Fig. S7A–E). Short and medium chain fatty acids were decreased after ozone exposure in all offspring (females > males) regardless of diet (Supplementary Fig. S7A). Increases in circulating polyunsaturated fatty acids (PUFAs, i.e., docosahexaenoate [DHA; 22:6n3] and arachidonate [20:4n6]) were observed in ozone-exposed male offspring from CD dams when compared to filtered air controls (Supplementary Fig. S7A). Interestingly, in the female HFD offspring group exposed to ozone, decreases were noted in diet-derived PUFAs (i.e., linoleate (18:2n6) and linolenate [alpha or gamma; (18:3n3 or 6)]) and long chain fatty acids as compared to the air-exposed group (Supplementary Fig. S7A). This cohort was also characterized by significant declines in monounsaturated fatty acids including myristoleate (14:1n5) and palmitoleate (16:1n7) (Fig. 3A; Supplementary Fig. S7A). In addition, the levels of several acylcarnitines (i.e., myristoylcarnitine [C14] and palmitoylcarnitine [C16]) were lower in all ozone-exposed males from CD dams and female offspring from both dietary groups, with the most pronounced effects observed for females from HFD-fed dams (Fig. 3B,C; Supplementary Fig. S7B). This was associated with decreases in 3-hydroxy fatty acids (i.e., 3-hydroxylaurate and 3-hydroxymyristate) in ozone vs air female offspring regardless of maternal diet (Supplementary Fig. S7C). These compounds are intermediates of beta-oxidation (Fig. 3D; Table 3). Elevated levels of odd chain dicarboxylates (i.e., pimelate (C7-DC) and azelate (C9-DC)) were observed in all ozone-exposed male and female groups, while even chain dicarboxylates were lower only in ozone-exposed female groups regardless of diet (Fig. 3A). Since the metabolism of odd chain and even chain dicarboxylates is compartmentalized, and odd chain dicarboxylates feed into beta-oxidation while even-chain species are suggested to supply the TCA cycle (Fig. 3D; Table 3).

**Table 3:** Summary of major ozone-related changes in serum metabolites in young male and female offspring from dams fed control diet (CD) or high-fat diet (HFD).

Female ozone effect	Male ozone effect	Interpretation
**Lipid metabolism**		
↓ in diet-derived PUFAs (i.e., linoleate and linolenate) in HFD group ↓ in short and medium chain fatty acids in both CD and HFD and in long-chain fatty acids in HFD groups ↓ in acyl carnitines (HFD > CD) ↓ in hydroxyl fatty acids ↑ in odd chain decarboxylates ↓ in even chain decarboxylates	↑ in PUFAs (i.e., DHA and arachidonate) (CD > HFD) ↓ in short and medium chain fatty acids (fewer than females) in both CD and HFD ↓ in acyl carnitines only in CD group ↑ in odd chain decarboxylates	↑ in breakdown of membrane phospholipids ↓ in desaturase activity ↓ in beta-oxidation (mitochondria) ↑ in omega-oxidation (peroxisomes) Mitochondrial impairment and compensation by omega-oxidation in peroxisomes Sex-specific differences in ozone effects on beta-oxidation and TCA cycle
**Energy metabolism**		
→ in glucose ↑ in pyruvate ↑ in TCA cycle intermediates (HFD > CD)	↑ in glucose	↑ in glucose production and/or ↓ in insulin secretion in males after ozone exposure ↑ in glycolysis and TCA cycle in females after ozone exposure (HFD > CD)
↑ in BCAA catabolites ↑ in ethylmalonate	↑ in BCAA catabolites	↑ use of BCAA for energy and/or impairment in mitochondrial function in both sexes after ozone exposure
**Nitrogen balance**		
↑ in acetylated amino acids (HFD > CD) ↑ in urea in CD ↑ in polyamines and MTA (HFD > CD)	↑ in acetylated amino acids (HFD > CD) ↑ in urea in the HFD group ↑ in polyamines	↑ in protein degradation by ozone exposure Alternations in nitrogen balance (HFD > CD) ↑ in activation of protective mechanisms (DNA stability, cell differentiation, and proliferation)
**Tryptophan and NAD metabolism**		
↑ in kynurenine and downstream metabolites	↑ in kynurenine and xanthurenate in HFD group	Kynurenine, produced from tryptophan, serves to downregulate inflammation and is an intermediate for NAD synthesis, thus increases might indicate increased energetics and signal transduction
**Steroid metabolism**		
↑ in 7-HOCA and 7-hydroxycholesterol ↓ pregnanolone/ allopregnanolone sulfate	↑ in 7-HOCA	Ozone may affect a P450 family of enzymes involved in steroid metabolism and change bile acids synthesis Differences in females’ progestin steroids may suggest ozone effect on hypothalamic–pituitary–adrenal axis
↓ Biliverdin and bilirubin (CD > HFD)	↓ Biliverdin and bilirubin (CD > HFD)	Reactive oxygen species levels are likely high in ozone-exposed rats (CD)
**Microbiome**		
↑ Benzoate metabolites (carboxylates) by ozone (HFD > CD)		Potential change in gut microbiome after ozone exposure in females

Data are summarized from the metabolomics report on ozone-induced changes in serum metabolites in young male and female offspring (n = 8/group) from dams that were fed CD or  HFD. ↑ indicates increase in metabolite levels, ↓ indicates decrease in metabolite levels, and → indicates no change in metabolite levels.

*PUFAs* polyunsaturated fatty acids, *TCA cycle* tricarboxylic cycle, *BCAA* branched-chain amino acids, *MTA* methylthioadenosine, *NAD* nicotinamide adenine, *7-HOCA* 7-alpha-hydroxy-3-oxo-4-cholestenoate.

Ozone exposure-related changes were noted in phospholipid metabolism. These were characterized by decreases in phosphatidylcholines in male and female offspring from both dietary groups and increases in phosphatidylethanolamines and phosphatidylinositols in females from HFD dams (Supplementary Fig. S7D). Ozone also had marked effects on lysophospholipid subclasses in both sexes from CD and HFD dams (Supplementary Fig. S7D). Monoacylglycerols were increased in ozone-exposed male and female offspring from CD whereas diacylglycerols were decreased in female offspring from CD dams (Supplementary Fig. S7E). Sphingolipid metabolites were increased primarily in ozone-exposed female offspring from CD dams (Supplementary Fig. S7E).

**Figure 4:**
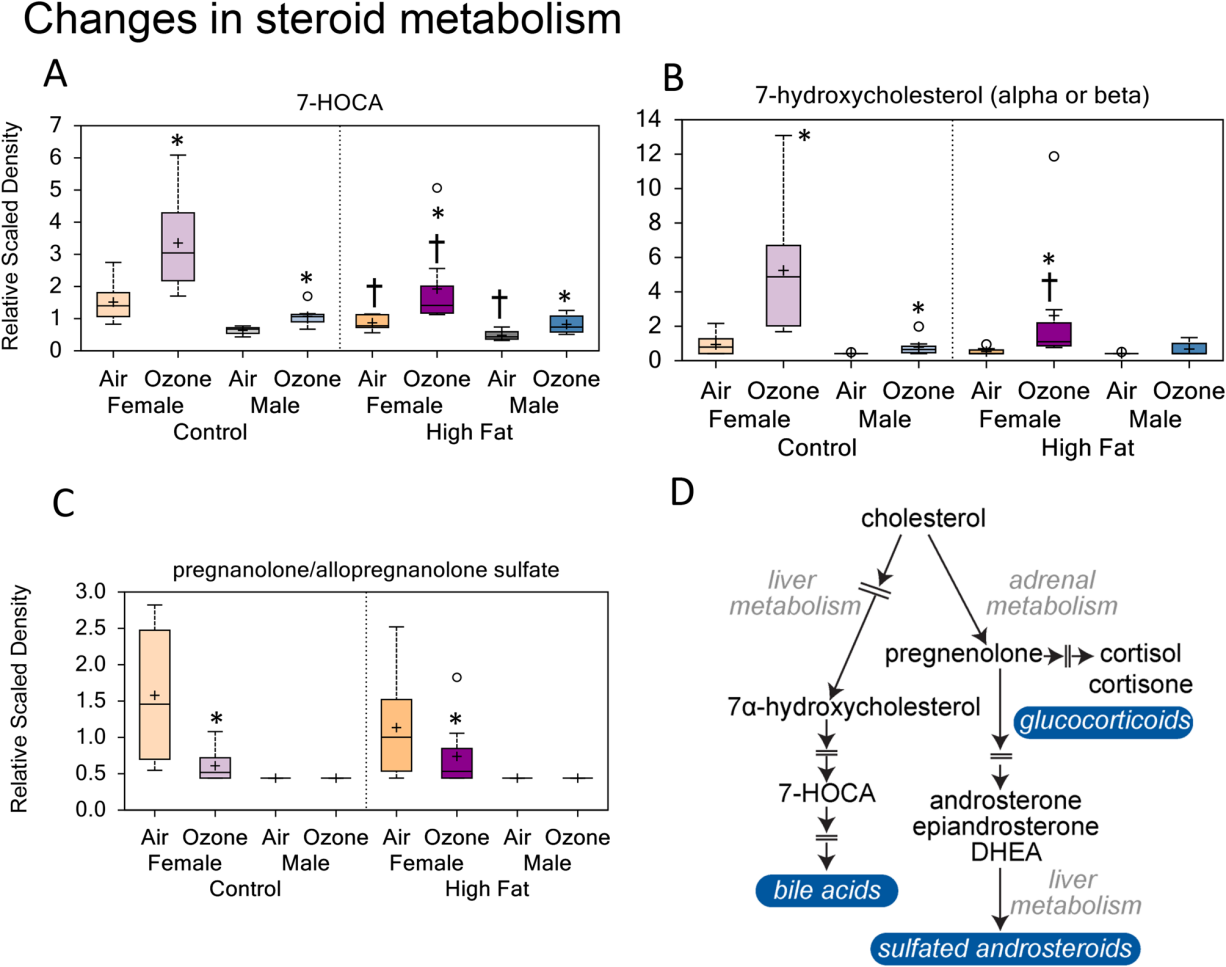
Ozone-induced changes in serum metabolites indicative of alterations in steroid metabolism in young male and female offspring from dams fed control diet (CD) or high-fat diet (HFD). Box plots (n = 8/group) showing ozone-induced changes in serum 7-alpha-hydroxy-3-oxo-4-cholestenoate (7-HOCA) (**A**), 7-hydroxycholesterol (**B**), and pregnanolone/allopregnanolone sulfate (**C**). *Significant effect of ozone exposure (p < 0.10 or p < 0.05) when compared to matching air group. ^†^Significant effect of maternal HFD (p < 0.10 or p < 0.05) when compared to matching maternal CD group. Schema of cholesterol metabolism leading to bile acid and steroids synthesis (**D**).

Although no major changes were noted in serum overall cholesterol levels determined through clinical analysis (Supplementary Fig. S5), marked increases in 7-alpha-hydroxy-3-oxo-4-cholestenoate (7-HOCA) were observed in response to ozone exposure in male and female offspring from both CD and HFD dams (Fig. 4A; Supplementary Fig. S7E). In addition, 7-hydroxycholesterol (alpha or beta) was significantly elevated in female offspring exposed to ozone regardless of diet (Fig. 4B; Supplementary Fig. S7E). In contrast, pregnanolone/allopregnanolone sulfate was lower in female offspring exposed to ozone compared to those exposed to air (Fig. 4C). Taken together, these changes suggest the effect on a P450 family of enzymes involved in steroid metabolism (Fig. 4D; Table 3).

### Alterations in energy metabolism

**Figure 5:**
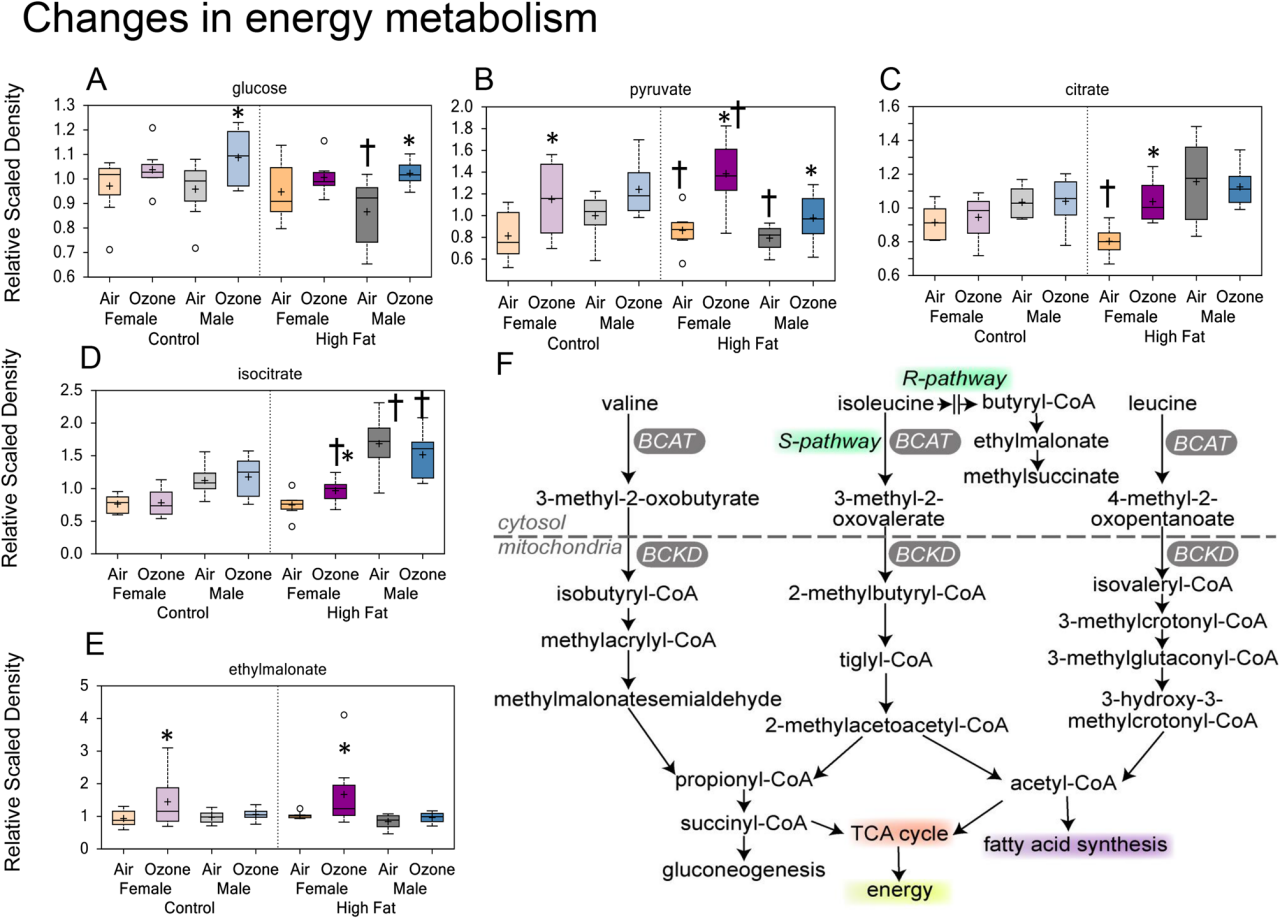
Maternal high-fat diet (HFD)-induced changes in serum metabolites involved in energy metabolism in young male and female offspring: the effect of acute ozone exposure. Boxplots showing ozone-induced changes in serum glucose (**A**), pyruvate (**B**), citrate (**C**), isocitrate (**D**), and ethylmalonate (**E**). *Significant effect of ozone exposure (p < 0.1 or p < 0.05) when compared to matching air group. ^†^Significant effect of maternal HFD (p < 0.1 or p < 0.05) when compared to matching maternal control diet (CD) group. Schema of branched-chain amino acid metabolism and their use in energy production through TCA cycle (**F**).

Ozone exposure increased circulating glucose levels in male offspring (Fig. 5A) as we have noted in our previous studies in male Wistar Kyoto rats^23^. These differences were not observed in ozone-exposed female offspring. However, pyruvate involved in glycolysis was elevated in females (Fig. 5B). Consequently, TCA cycle intermediates (i.e., citrate and isocitrate) were higher in female offspring from HFD dams exposed to ozone (Fig. 5C,D; Supplementary Fig. S8). Ozone-induced increases in branched-chain amino acid (BCAA) catabolites, isovalerylcarnitine (C5) and isobutyrylcarnitine (C4), in male and female offspring from CD and HFD dams (Supplementary Fig. S9A). Interestingly, accumulation of ethylmalonate was increased in ozone-exposed female offspring from CD and HFD dams (Fig. 5E). These changes may indicate increased utilization of BCAA for energy production (Fig. 5F; Table 3).

### Changes in nitrogen balance

**Figure 6:**
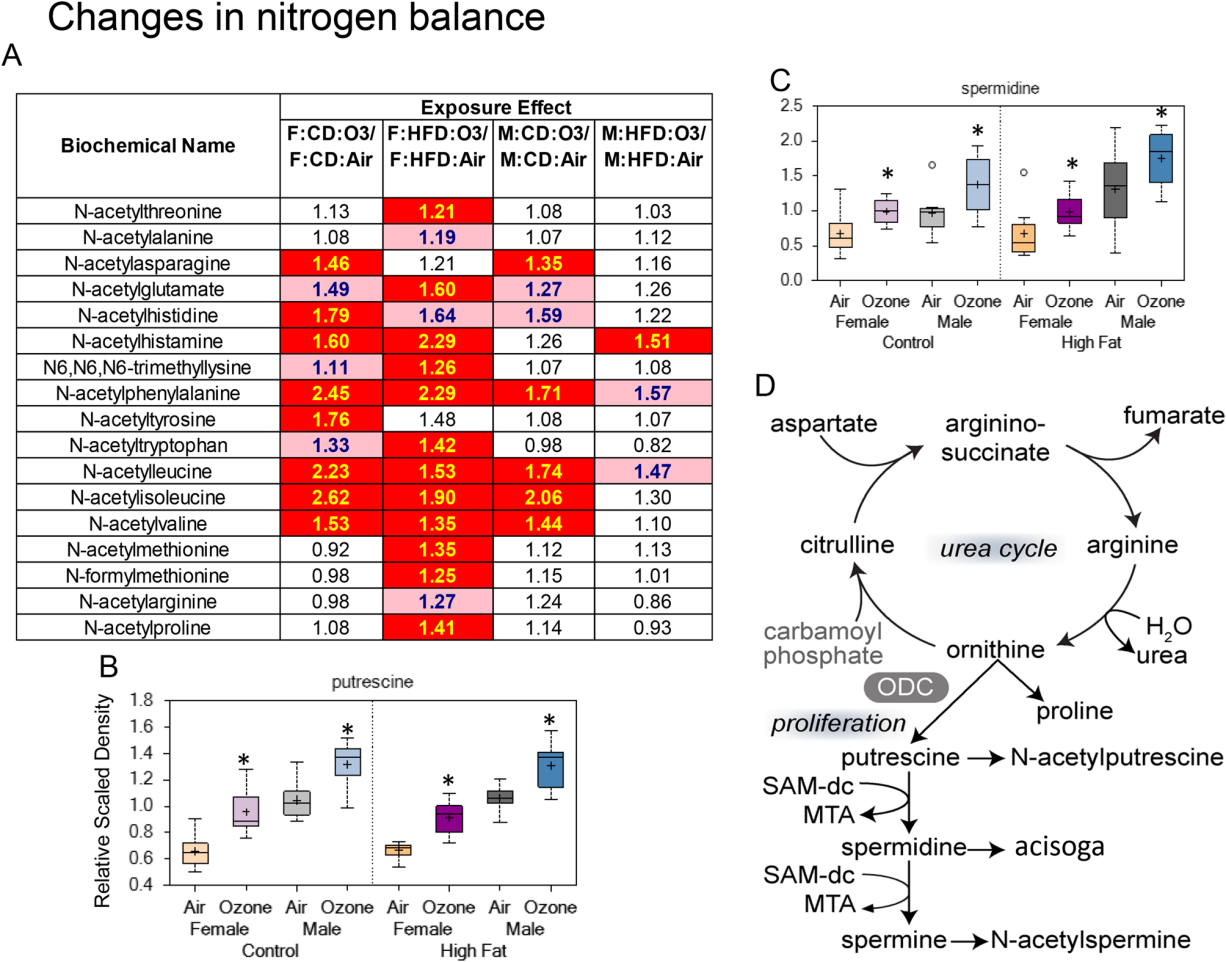
Maternal high-fat diet (HFD)-induced changes in serum acetylated amino acids, and polyamines indicating shift in nitrogen balance in young male and female offspring: the effect of acute ozone exposure. Heat map showing ozone-induced changes in acetylated amino acids (**A**). The values show mean fold change due to ozone exposure when compared to filtered air in offspring. Red indicates p ≤ 0.05; pink indicates p ≤ 0.1 to p ≤ 0.05. Box plots showing ozone-induced changes in serum putrescine (**B**), and spermidine (**C**). *Significant effect of ozone exposure (p < 0.1 or p < 0.05) when compared to matching air group. ^†^Significant effect of maternal HFD (p < 0.1 or p < 0.05) when compared to matching maternal control diet (CD) group. Schema of polyamine metabolism and urea cycle (**D**).

There were major ozone exposure-related changes, most of which were increases, in various metabolites involved in protein metabolic processes (Supplementary Fig. S9A,B). These changes were associated with general increases in metabolites associated with glycine, glutamate, histidine, tryptophan, leucine, isoleucine, valine, methionine, and polyamine metabolism. These changes occurred primarily in female offspring (HFD > CD) (Fig. 6A). Some tryptophan, leucine, and valine metabolites were also increased in male offspring from CD and HFD dams (Fig. 6A). The changes in acetylated amino acids were accompanied by an ozone-induced increase in urea in offspring from HFD dams (Supplementary Fig. S9B; significant when both sexes combined), suggesting alterations in nitrogen balance. In addition, polyamine levels (i.e., putrescine and spermidine) were elevated for all ozone versus air comparisons (Fig. 6B,C; Supplementary Fig. S9B). Collectively, changes in acetylated amino acids and urea could point towards increased protein degradation in ozone-exposed animals from HFD dams (Fig. 6D; Table 3).

### Perturbations in tryptophan and nicotinamide adenine dinucleotide (NAD) metabolism

**Figure 7:**
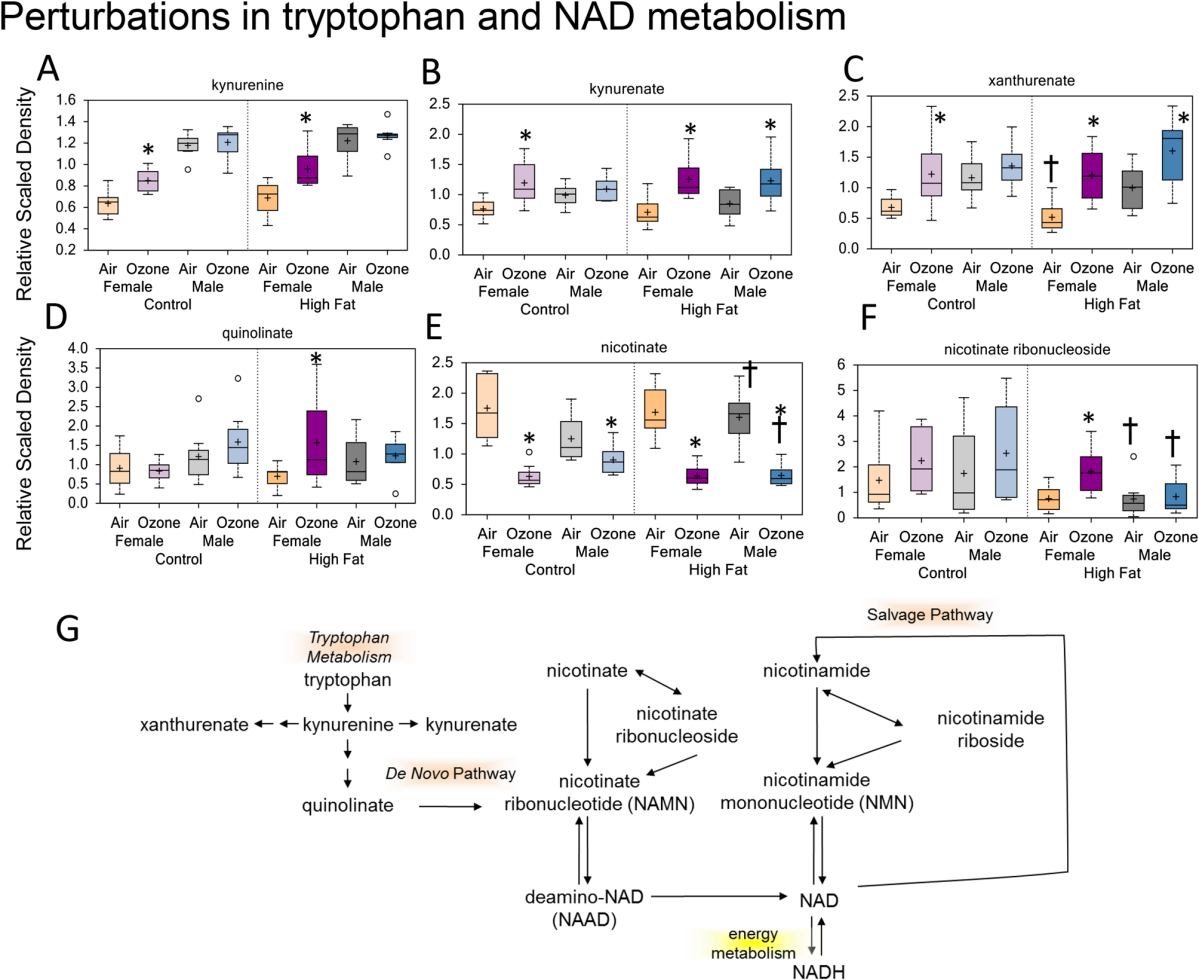
Maternal high-fat diet (HFD)-induced perturbations in tryptophan and nicotinamide adenine dinucleotide (NAD) metabolism in young male and female offspring: the effect of acute ozone exposure. Box plots showing ozone-induced changes in serum kynurenine (**A**), kynurenate (**B**), xanthurenate (**C**) quinolinate (**D**), nicotinate (**E**), and nicotinate ribonucleoside (**F**). *Significant effect of ozone exposure (p < 0.1 or p < 0.05) when compared to matching air group. ^†^Siignificant effect of maternal HFD (p < 0.1 or p < 0.05) when compared to matching maternal control diet (CD) group. Schema of tryptophan and NAD metabolism (**G**).

Elevated levels of kynurenine and its downstream metabolites, including kynurenate, xanthurenate, and quinolinate, were observed in ozone-exposed female offspring from both CD and HFD dams as compared to air-exposed animals (Fig. 7A–D). Kynurenate (Fig. 7B) and xanthurenate (Fig. 7C) were also elevated in ozone-exposed male offspring from HFD dams. These increases were associated with decreases in nicotinate in ozone-exposed female and male offspring from both dietary groups (Fig. 7E). Ozone exposure increased nicotinate ribonucleoside in female offspring from HFD dams (Fig. 7F). These metabolites are involved in the NAD salvage pathway (Fig. 7G).

### Alterations in gut microbiome-associated compounds

**Figure 8:**
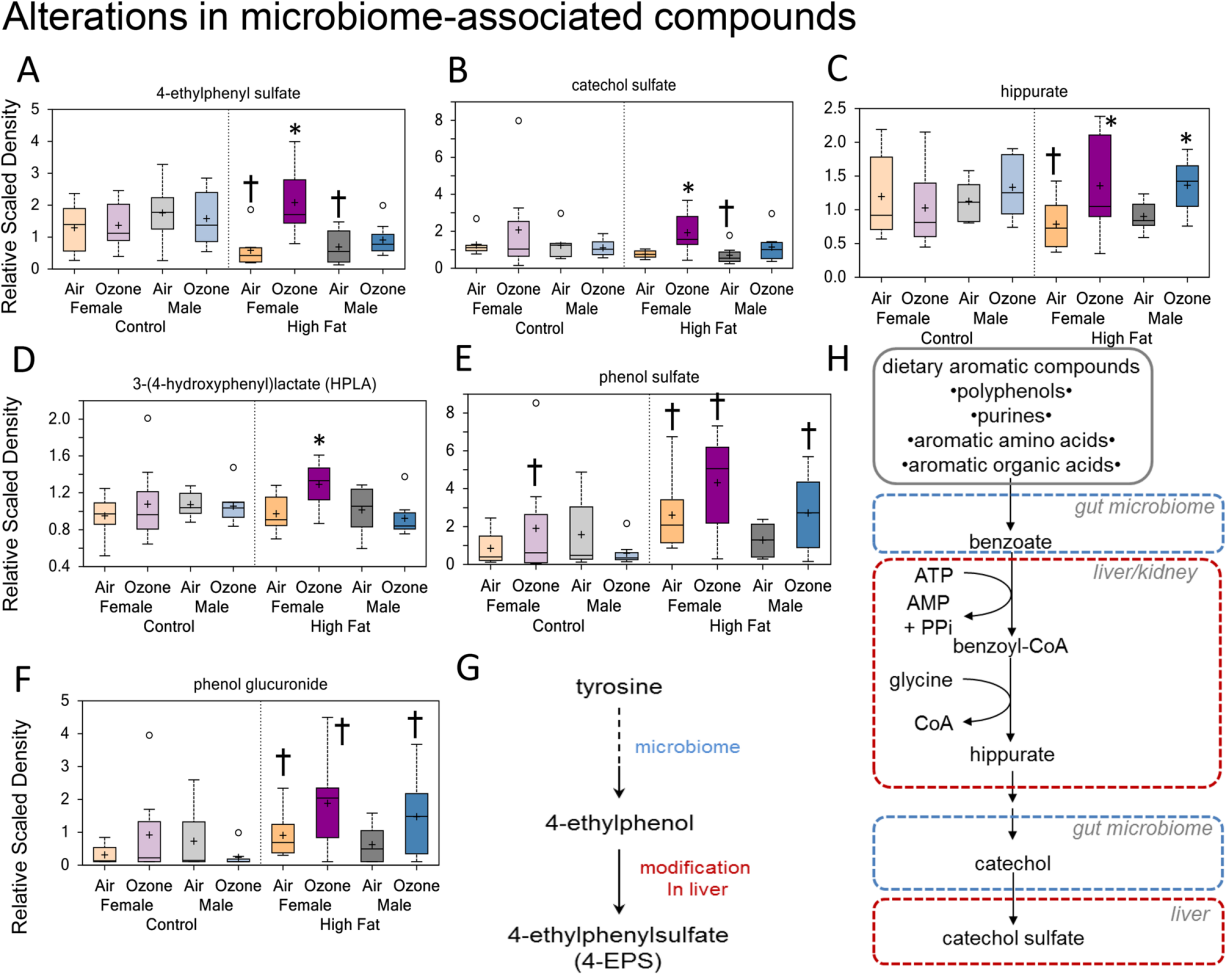
Maternal high-fat diet (HFD)-induced changes in serum metabolites reflective of alterations in gut microbiome in young male and female offspring: the effect of acute ozone exposure. Box plots showing ozone-induced changes in serum 4-ethylphenyl sulfate (**A**), catechol sulfate (**B**), hippurate (**C**), 3-(4-hydroxyphenyl) lactate (**D**), phenol sulfate (**E**), and phenol glucuronide (**F**). *Significant effect of ozone exposure (p < 0.1 or p < 0.05) when compared to matching air group. ^†^Significant effect of maternal HFD (p < 0.1 or p < 0.05) when compared to matching maternal control diet (CD) group. Schematics of how microbiome can influence the production of 4-ethylphenyl sulfate from tyrosine (**G**), and how gut microbiome can change production of amino acid intermediates to produce catechol sulfate (**H**).

Ozone-induced changes were noted in several biochemicals involved in gut microbial metabolism. In contrast to HFD-induced decreases in air-exposed offspring (Supplementary Fig. S6), the levels of benzoate metabolites (i.e., 4-ethylphenylsulfate, catechol sulfate, and hippurate) were elevated in ozone-exposed females from HFD dams (Fig. 8A–C). Benzoate metabolites are simple carboxylic acids produced from the microbial degradation of dietary aromatic compounds in the intestine, such as polyphenols, purines, and aromatic organic acids. In addition, microbiome-contributed tyrosine metabolite, 3-4-hydroxyphenyl lactate, was increased in the ozone-exposed female offspring from HFD dams (Fig. 8D). Moreover, ozone exposure tended to increase microbiome catabolites of tyrosine, phenol sulfate (Fig. 8E) and phenol glucuronide (Fig. 8F), in both female and male offspring from HFD dams. Collectively, these changes show ozone-induced changes in markers of enteric microbiome in male and female offspring from HFD dams (Fig. 8G,H; Table 3).

## Discussion

Obesity and HFD during pregnancy have been linked to developmental reprogramming of metabolic processes in the offspring within the hypothalamus through neuroendocrine pathways^32,33^. Often, the impacts of developmental stressor exposures or maternal HFD-induced obesity are amplified when the offspring are challenged with environmental or non-chemical stressors^32^. We postulated (1) maternal HFD and obesity will predispose male and female offspring to systemic metabolic abnormalities, that of which can be exacerbated when offspring are exposed acutely to an environmental air pollutant stressor (e.g., ozone) and (2) comprehensive serum neuroendocrine and metabolic hormone assessment, together with global metabolomics, will delineate possible metabolic risk factors in offspring due to maternal high fat diet and obesity. Although we found no major maternal diet-related or ozone-induced changes in circulating neuroendocrine or metabolic hormones, alterations in steroidal metabolites and increases in BCAA were predictive of involvement of the hypothalamic–pituitary–adrenal axis in ozone-induced metabolic impairments. We found that maternal HFD in air-exposed offspring altered circulating lipids in a sex-specific manner (males > females), and decreased levels of anhydroglucitol in males, indicating a potential susceptibility to insulin resistance. Maternal HFD in air-exposed offspring decreased levels of benzoate metabolites linked to gut microbiome in both sexes. When exposed to ozone, many types of lipid metabolites were altered, including PUFAs, FFA, acyl carnitines, and gycerols in a sex-specific manner, suggesting lipid redistribution in both sexes (females > males) and FFA mobilization in males. Major changes in nitrogen balance, including tyrosine and tryptophan metabolites, and increases in BCAA after ozone exposure were suggestive of alterations in preference for energy source (females > males). Female offspring were also more sensitive to ozone-induced changes in metabolites related to gut microbiota. Ozone-induced changes in amino acid metabolites involved in tryptophan metabolism, and metabolites that regulate autophagy and inflammation in various chronic diseases including neuropsychiatric conditions^34,35^ in offspring from HFD dams suggests their greater susceptibility to chronic disorders in adulthood. Thus, maternal HFD, while increasing susceptibility of offspring to metabolic and chronic diseases, can impair the response to environmental stressors in a sex-specific manner.

LE rats have been widely used for developmental and neurobehavioral research including developmental environmental exposures^36,37^. These rats are also sensitive to diet-induced metabolic changes^38^. We have shown that females given HFD prior to and during pregnancy are obese and have offspring with increased body mass and body fat %^29,30^. Moreover, male adult offspring (PND 150–170) appear more susceptible to ozone-induced pulmonary injury and inflammation than females^29^. Since peri-adolescent rats are susceptible to neuroendocrine stress-mediated metabolic alterations ^39^, we selected this age in our current work. Moreover, this also represents a period when children spend more time outdoors and thus, likely encounter highest exposures to air pollutants, including ozone. In the present study, we noted that diet-induced obesity in dams was translated to obesity in male and female offspring at a young age of PND 40 (females > males). Furthermore, female offspring from obese HFD dams, at the time of the onset of puberty, were more susceptible to acute ozone-induced lung inflammation and systemic metabolic alterations.

In order to determine systemic metabolic alterations and to predict changes in metabolically active peripheral organs in our model, we used a global metabolomic approach. We have shown that serum metabolite profiling is useful in characterizing acute stress-mediated homeostatic metabolic alterations in peripheral organs, such as liver, adipose tissue, and muscle tissue^23^. Increases in circulating FFA, BCAA, and intermediates of the TCA cycle suggested adipose lipolysis, muscle protein catabolism, increased gluconeogenesis, and changes in liver mitochondrial function after ozone exposure in male Wistar Kyoto rats^23^. These ozone-induced glucose, lipid and protein metabolic changes were apparent without peripheral insulin resistance but impairment in pancreatic insulin secretion in response to glucose injection^25^. We have also shown increases in circulating FFA and membrane phospholipids in humans after ozone exposure^27^.

To understand how maternal diet may alter susceptibility to ozone-induced metabolic alterations in offspring, it is critical to first determine how maternal HFD induces changes in the profile of circulating metabolites in offspring exposed to air. In the Wistar rat model, maternal obesogenic diet was associated with increased adiposity index, triglycerides, and insulin as well as multiple changes in the liver that are similar to non-alcoholic fatty liver disease in male offspring but not in female offspring^13^. In the present study involving LE rats, although the metabolic impairment due to maternal HFD was not associated with increased insulin or circulating triglycerides, many lipid metabolites were affected in a sex-specific manner (males > females). Decreased anhydroglucitol in male offspring may indicate insulin resistance predisposition as reported by Lomas-Soria and colleagues^13^. Although at younger age of 40 day, we did not see changes in circulating insulin due to maternal HFD or ozone, our previous study has shown increases in ozone-induced glucose intolerance in male offspring from HFD dams at an older age of 150–170 days^29^. The metabolomic data also suggested that maternal HFD might impact the offspring’s gut microbiome since benzoate metabolites were markedly decreased in both female and male offspring. The influence of maternal diet on the gut-brain axis, pregnancy-related changes in the maternal hepatic portal circulation, and placental maladaptation have been proposed to influence the offspring’s gut microbiome^40,41^.

We have shown that acute ozone exposure induces systemic metabolic alterations through the activation of the neuroendocrine system^10,23–25,27^. In this study, we wanted to determine if maternal HFD altered ozone responsiveness to induce metabolic alterations in the offspring. Although we did not observe major maternal diet-related or ozone-induced changes in neuroendocrine or metabolic hormones in the offspring, since stress response is dynamic and some of these hormones may exhibit ultradian fluctuations^42^, the ozone effects on circulating metabolites, such as increases in BCAA in both sexes and long-chain fatty acids in males as well as changes in steroid metabolites, are consistent with stress hormone mediation of metabolic alterations^23,24^. In relation to ozone-induced changes in metabolites involved in other lipid metabolic processes in offspring, the effects were distinct from our previous study on male Wistar Kyoto rats^23^. Specifically, short chain, medium chain, and monohydroxy fatty acids as well as acyl carnitines were decreased, especially in ozone-exposed female offspring from HFD dams, unlike changes observed in male Wistar Kyoto rats. These differences suggest likely rat strain- and sex-related variations in how a stressor response may induce metabolic alterations and how maternal HFD may increase susceptibility of female offspring to metabolic syndrome. Changes in lipid metabolism in the current study involved many classes of membrane phospholipids as we have observed in a human clinical study where ozone exposure occurred during intermittent exercise^27^. Collectively, we show widespread ozone-induced changes in lipid metabolism that are influenced by maternal obesity and HFD (females > males), which, when challenged with a stressor, may disproportionately predispose female offspring to greater metabolic disorders later in life.

Omega-oxidation is an alternative to beta-oxidation, which takes place in peroxisomes and generates dicarboxylic fatty acids^43^. Elevated levels of odd chain dicarboxylates in ozone-exposed male and female offspring from both dietary groups, and lower levels of even chain dicarboxylates in females could point towards decreased beta-oxidation resulting from altered mitochondrial function and an attempt to compensate by increases in omega-oxidation. Since the metabolism of odd chain and even chain dicarboxylates is compartmentalized and odd chain dicarboxylates feed into beta-oxidation while even-chain species supply TCA cycle^44^, the changes observed here may suggest differential mitochondrial effects of ozone in male and female offspring. The absence of ozone-induced increases in glucose together with increases in pyruvate and TCA cycle intermediates in female offspring might indicate sex differences in metabolite preference for energy production in response to ozone. Decreases in acyl carnitines have been associated with progression of Alzheimer’s disease in humans^45^.

Deficiency of carnitine acetyltransferase, an enzyme that buffers the mitochondrial acetyl-CoA pool by converting short-chain acyl-CoAs to their membrane permeant acylcarnitine counterparts, has been linked to diet-induced metabolic diseases^46^. Ozone exposure increased several acetylated amino acid species, with the highest number of significant changes detected in females from HFD dams. These increases may indicate ozone-induced mitochondrial stress in tissues, that is exacerbated by maternal HFD in female offspring. Collectively, these changes in acetylated amino acids along with increases in urea levels in ozone-exposed offspring from HFD dams indicate alterations in nitrogen balance and mitochondrial dysfunction, and suggest maternal obesity and diet disproportionately affect female offspring.

Polyamines play a critical role in the regulation of nucleic acid stability, cell differentiation and proliferation, and tissue repair^47^. Ozone exposure in the current study increasedcirculating polyamines (i.e., putrescine and spermidine) in female and male offspring from CD and HFD dams. An increase in 5-methylthioadenosine (MTA) in ozone-exposed female offspring from HFD dams is consistent with increased rates of polyamine synthesis, which showed the greatest effect. The source of increased polyamines in the circulation cannot be ascertained from the current experiment, however, the lung plays an important role in polyamine metabolism^48^. Furthermore, ozone exposure has been shown to increase putrescine and spermidine in the lung^49^, suggesting the role of circulating polyamines in systemic and pulmonary effects.

Kynurenine is produced from tryptophan in response to inflammatory stimuli and has been linked to many disease conditions^50–52^. Ozone exposure was associated with increases in kynurenine and its downstream metabolites. Since kynurenine is also an intermediate in de novo NAD^+^ synthesis from tryptophan, these changes suggest that ozone exposure causes an increased demand for NAD^+^. This was further supported by ozone-induced decreases in nicotinate in all experimental groups and increases in nicotinate ribonucleoside in female offspring from HFD dams, which are involved in the NAD salvage pathway. Taken together, changes in the kynurenine pathway may be linked to activation of inflammatory responses and could also contribute to de novo NAD synthesis. Changes in NAD availability may impact both energetic and signal transduction processes. Female offspring, which showed greater neutrophilic inflammation than males in this study, seem to be more affected by these ozone effects.

In addition to maternal HFD, ozone exposure also impacted gut microbiome metabolites in female offspring from HFD dams, especially those resulting from benzoate metabolism. Maternal HFD, obesity, and stress have been linked to altered gut microbiome^53–55^. Benzoate metabolites are simple carboxylic acids produced from the microbial degradation of dietary aromatic compounds in the intestine, such as polyphenols, purines, and aromatic organic acids. These changes suggest that acute ozone exposure can modify the activity of enteric microbiome with female offspring from HFD dams being more sensitive to these effects. The increases in 7-HOCA after ozone exposure might also be linked to gut microbiome changes since gut microbes are involved in production of secondary bile acids and 7-HOCA is an intermediate in this process^56^. Gut microbiome changes due to maternal diet and acute ozone exposure may underlie the marked changes in brain and neuroendocrine pathways after ozone exposure^10,57^.

While acute ozone inhalation was used as a stressor to unmask the potential influence on susceptibility of adolescent offspring to metabolic disorders as a result of maternal HFD, long term exposures to environmental stressors will be necessary to reveal the contribution of chronic stress to metabolic disease. Importantly, there are likely species-related and/or genetic differences in sensitivity to diet-induced obesity that were not considered in our current work. Nonetheless, we examined susceptibility of 40-day old offspring, the time of sexual maturity (puberty) in male and female rats, to the air pollutant ozone. This age may be a susceptible window to air pollutants because adolescent children spend significant time outdoors, encountering higher concentrations of ozone relative to adulthood. Although, systemic changes in metabolites are predictive of changes in metabolically active organs, we did not assess specific organ effects of maternal HFD or acute ozone exposure in offspring.

Collectively, we report here that maternal HFD in air-exposed offspring is associated with obesity together with changes in circulating lipid profile and benzoate metabolites, suggestive of changes in gut microbiome in both male and female offspring (male > female). These offspring when exposed to a challenge environmental stressor, such as ozone, have more profound changes in a number of metabolic processes involving lipid, protein, and energy metabolism, indicative of impairment of mitochondrial function, susceptibility to insulin resistance, and alterations in gut microbiome. Female offspring from HFD dams are more affected by ozone stressor then males. Some of the ozone-induced changes in metabolic processes are suggestive of mediation through neuroendocrine pathways involving stress hormones. Thus, maternal obesity and HFD disproportionately predisposes female offspring to greater systemic metabolic alterations when challenged with an environmental stressor. These data provide insights into how maternal HFD and obesity might result in sex-specific metabolic disorders through complex interactions between multiple key metabolic pathways when challenged with environmental stressors.

## Materials and Methods

### Animals and feeding regimen

The dietary regimen followed our previously reported protocol^29^. Long-Evans (LE) female rats, 30-day old (post-natal day (PND) 30), were fed either a control diet (CD; 10% of calories from fat; TD.08806) or high-fat diet (HFD; 60% of calories from fat; TD.06414) from Harlan Laboratories (Teklad Custom Research Diets; Madison, WI) at Charles River Laboratories (Raleigh, NC). The diet composition is provided in the Supplemental Materials (Supplementary Table S1) and in a previous publication^29^. A 60% HFD was used to induce obesity in this model as female rats display an increase in resistance to HFDs compared to males^58^. Rats were bred at PND 72 and shipped to our animal facility that is approved by the Association for Assessment and Accreditation of Laboratory Animal Care, on gestational day (GD) 1. Dams continued CD and HFD feeding throughout pregnancy and until weaning at PND 21. Two offspring of each sex were randomly chosen from each litter for this study (n = 10 pregnant dams/dietary regimen, 1 pup/sex/litter/exposure, litter was the unit for offspring). At PND 30, all offspring were fed CD. Protocols for ethical use of animals were approved by the United States Environmental Protection Agency’s Institutional Animal Care and Use Committee. All experiments were performed in accordance with the National Institute of Health Guide lines for the care and use of laboratory animals (https://grants.nih.gov/grants/olaw/guide-for-the-care-and-use-of-laboratory-animals.pdf).

### Body composition measurements

Body weight and composition were assessed as previously described^29^. Briefly, maternal body weight was assessed on GD 1, 7, 10, 14, 17, and 21 and body composition was analyzed on GD 7 and 21. Offspring body weight and body composition measurements were taken on PND 37. This analysis employed a Bruker Minispec LF90 II TD-NMR body composition analyzer (Bruker Optics, Inc., Billerica, MA, USA) to estimate body fat %, lean body mass %, and body fluid %.

### Ozone exposure

We chose adolescent (PND 40) age for rat to represent the peri-adolescent age in humans, when time spent outdoors is maximum and likely will result in the highest ozone exposure response. At PND 40, female and male offspring were exposed to either filtered air or ozone (0.8 ppm) for 5 h (n = 10/group) in Rochester style “Hinners” chambers. As reported earlier^30^, ozone was generated from oxygen using a silent arc discharge generator (OREC, Phoenix, AZ) and transported to the exposure chambers. To avoid diurnal variation in biological endpoints, all staggered exposures occurred between 7 am and 11 am immediately followed by necropsy within 2 h. The air temperature, relative humidity, and ozone concentrations [mean ± standard error of mean (SEM)] for the air and ozone exposure chambers are as follows: filtered air chamber: 72.44 ± 0.14 °C, 52.72 ± 0.22%, 0.0 ± 0.0 ppm, respectively; ozone chamber: 73.84 ± 0.12 °C, 51.09 ± 0.22%, 0.7999 ± 0.0016 ppm, respectively. This concentration of ozone is several folds higher than what may be encountered in the environment. However, it has been shown that the lung dose received during human clinical studies, where volunteers are exposed to 0.2–0.4 ppm ozone during intermittent exercise is comparable to rodents exposed to 0.8–1.0 ppm during their inactivity^59^. Since children playing outdoors in sun light might deposit high ozone dose, the concentration we used in resting young rodents is justifiable.

### Necropsy, blood sample collection, and lavage fluid analysis

Within 2 h following air or ozone exposure, rats were euthanized with an overdose of sodium pentobarbital (> 200 mg/kg of Fatal Plus, Vortech Pharmaceuticals, Ltd., Dearborn, MI). Blood samples were collected from the abdominal aorta in two vacutainer tubes; one containing EDTA as an anticoagulant to obtain plasma and a second serum separator tube to obtain serum samples^30^. Plasma and serum samples were prepared by centrifugation and stored at − 80 °C for further processing. The lungs were lavaged to collect bronchoalveolar lavage fluid (BALF) as previously described^30^. BALF total cell count, cell differentials, and injury marker levels [total protein, albumin and n-acetylglucosaminidase (NAG) activity] were assessed as previously described^22,31^.

### Clinical assessment of circulating hormones and metabolites

Serum metabolic markers [total cholesterol, high-density lipoprotein (HDL) cholesterol, low-density lipoprotein (LDL) cholesterol, triglycerides, and free fatty acids (FFA)] were separately analyzed using clinical assays as previously described^24^. Serum levels of adrenocorticotropic hormone (ACTH), brain-derived neurotrophic factor (BDNF), follicle stimulating hormone (FSH), growth hormone (GH), prolactin (PRL), luteinizing hormone (LH), and thyroid stimulating hormone (TSH) were analyzed using rat-specific multianalyte assessment using Millipore reagents and kit (Millipore Inc., Burlington, MA, USA). Plasma epinephrine levels were quantified using a kit obtained from Rocky Mountain Diagnostics following the manufacturer’s protocol (Colorado Springs, CO, USA) and serum corticosterone levels were assessed using a kit from Arbor Assays (Ann Arbor, MI, USA). Serum samples were analyzed for leptin using rat-specific electrochemiluminescence assay (Meso Scale Discovery, Gaithersburg, MD, USA) and insulin levels using rat-specific kits (Crystal Chem Inc., Elk Grove Village, IL, USA).

### Metabolomic assessment of serum samples

#### Sample preparation

Global serum metabolic profiles were determined for air- or ozone-exposed male and female offspring born to dams that received CD or HFD (n = 8/group). All procedures listed below for metabolomic analysis have been described previously^60^. Briefly, samples were prepared using the automated MicroLab STAR system from Hamilton Company. Several recovery standards were added to serum samples prior to the first step in the extraction process for QC purposes. Proteins were precipitated with methanol under vigorous shaking for 2 min (Glen Mills GenoGrinder 2000) followed by centrifugation as described previously^23,60^. The resulting extract was divided into five fractions: two for analysis by two separate reverse phase (RP)/UPLC-MS/MS methods with positive ion mode electrospray ionization (ESI), one for analysis by RP/UPLC-MS/MS with negative ion mode ESI, one for analysis by hydrophilic interaction liquid chromatography (HILIC)/UPLC-MS/MS with negative ion mode ESI, and one sample was reserved for backup. Samples were placed briefly on a TurboVap (Zymark) to remove the organic solvent. The sample extracts were stored overnight under nitrogen before preparation for analysis.

#### QA/QC

Based on the Metabolon Inc (Durham, NC) criteria^60^, several types of controls were analyzed in concert with the experimental samples: a pooled matrix sample generated by taking a small volume of each experimental sample served as a technical replicate throughout the data set; extracted water samples served as process blanks; and a cocktail of QC standards that were carefully chosen not to interfere with the measurement of endogenous compounds were spiked into every analyzed sample, allowed instrument performance monitoring and aided chromatographic alignment. Instrument variability was determined by calculating the median relative standard deviation (RSD) for the standards that were added to each sample prior to injection into the mass spectrometers. Overall process variability was determined by calculating the median RSD for all endogenous metabolites (i.e., non-instrument standards) present in 100% of the pooled matrix samples. Experimental samples were randomized across the platform run with QC samples spaced evenly among the injections. Procedures for instrument and process variability determinations were determined based on Metabolon Inc protocols (Durham, NC, USA) and have been reported previously ^60^.

#### Ultrahigh performance liquid chromatography–tandem mass spectroscopy (UPLC-MS/MS)

All methods utilized a Waters ACQUITY UPLC and a Thermo Scientific Q-Exactive high resolution/accurate mass spectrometer interfaced with a heated electrospray ionization (HESI-II) source and Orbitrap mass analyzer operated at 35,000 mass resolution have been reported previously^23,60^. Briefly, the sample extract was dried then reconstituted in solvents compatible to each of the four methods. Each reconstitution solvent contained a series of standards at fixed concentrations to ensure injection and chromatographic consistency. Based on Metabolon Inc protocols and previously published methods^60^, the first aliquot was analyzed using acidic positive ion conditions, chromatographically optimized for more hydrophilic compounds. The second aliquot was also analyzed using acidic positive ion conditions; however, it was chromatographically optimized for more hydrophobic compounds. The third aliquot was analyzed using basic negative ion optimized conditions and a separate dedicated C18 column. The fourth aliquot was analyzed via negative ionization following elution from a HILIC column (Waters UPLC BEH Amide 2.1 × 150 mm, 1.7 µm) using a gradient consisting of water and acetonitrile with 10 mM Ammonium Formate, pH 10.8. The MS analysis alternated between MS and data-dependent MS^n^ scans using dynamic exclusion. The scan range varied slighted between methods but covered 70–1000 m/z. Raw data files are archived and extracted as described below. These procedures have been published previously^60^ and explained in out prior publication^23^.

#### Data extraction and compound identification

Metabolon’s (Durham, NC, USA) hardware and software were used for data processing. Raw data were extracted, peak-identified, and QC checked. These systems are built on a web-service platform utilizing Microsoft’s .NET technologies, which run on high-performance application servers and fiber-channel storage arrays in clusters to provide active failover and load-balancing^60^. Compounds were identified by comparison to library entries of purified standards or recurrent unknown entities. Metabolon maintains a library based on authenticated standards that contains the retention time/index (RI), mass to charge ratio (*m/z*), and chromatographic data (including MS/MS spectral data) on all molecules present in the library. The biochemical identifications were based on three criteria: retention index within a narrow RI window of the proposed identification, accurate mass match to the library ± 10 ppm, and the MS/MS forward and reverse scores between the experimental data and authentic standards^60^. The MS/MS scores are based on a comparison of the ions present in the experimental spectrum to the ions present in the library spectrum. Based on one of these factors, it is likely that these molecules are similar and thus all three data points can be utilized to differentiate biochemicals. Currently, at Metabolon Inc, more than 3300 commercially available purified standard compounds have been acquired and registered into LIMS for analysis on all platforms.

#### Curation

A variety of curation procedures were carried out to ensure a high-quality data set for statistical analysis and data interpretation based on the protocols of Metabolon Inc. The QC and curation processes were designed to ensure accurate and consistent identification of true chemical entities, and to remove those representing system artifacts, mis-assignments, and background noise. Data visualization and interpretation was done using Metabolon Inc data analysts software to confirm the consistency of peak identification among various samples^60^. Each compound was matched with the library of metabolites and corrected if necessary. The data were log transformed and missing values, if any, were imputed (with the minimum observed value for each compound). Analysis of variance (ANOVA) contrasts were used to identify biochemicals that differed significantly between experimental groups. Biochemicals were considered significant when *p* ≤ 0.05 were achieved. Those approaching significance (0.05 < *p* < 0.10) were also identified. Three-way ANOVA was used to identify biochemicals exhibiting significant interaction and main effects for experimental parameters of diet, exposure, and sex. The false discovery rate (*q* value) was calculated for multiple comparisons.

### Statistical analysis of metabolic, hormone, and lung injury/inflammation data

We used Graphpad Prism v7.03 software (San Diego, CA, USA) for statistical analyses of clinical markers, lung injury/inflammation markers, as well as neuroendocrine and metabolic hormones data. These data for female and male offspring were analyzed separately using a two-way ANOVA with diet and exposure as the two factors and a Holm–Sidak post-hoc test (n = 10/group). A *p* value of < 0.05 was considered statistically significant.

## Supplementary information

Supplementary Information 1

## Abbreviations

7-HOCA: 7-Alpha-hydroxy-3-oxo-4-cholestenoate
ACTH: Adrenocorticotrophic hormone
ANOVA: Analysis of variance
BALF: Bronchoalveolar lavage fluid
BCAA: Branched-chain amino acids
BDNF: Brain-derived neurotrophic factor
CD: Control diet
DHA: Docosahexaenoate
ESI: Electrospray ionization
FA: Formic acid
FFA: Free fatty acids
FSH: Follicle stimulating hormone
GD: Gestational day
GH: Growth hormone
HILIC: Hydrophilic interaction liquid chromatography
HDL: High-density lipoprotein
HFD: High-fat diet
LDL: Low-density lipoprotein
LE: Long-Evans
LH: Luteinizing hormone
MTA: Methylthioadenosine
NAD: Nicotinamide adenine dinucleotide
NAG: N-Acetylglucosaminidase
PCA: Principle component analysis
PFPA: Perfluoropentanoic acid
PND: Post-natal day
PRL: Prolactin
PUFAs: Polyunsaturated fatty acids
QA: Quality assurance
QC: Quality control
RI: Retention time/index
RP: Reverse phase
RSD: Relative standard deviation
SEM: Standard error of mean
TCA cycle: Tricarboxylic acid cycle
TSH: Thyroid stimulating hormone
UPLC-MS/MS: Ultra high-performance liquid chromatography–tandem mass spectrometer

## References

[1] Craig M (2017). Prevalence of obesity among adults and youth: United States, 2015–2016. NCHS Data Brief..

[2] Razzoli M, Pearson C, Crow S, Bartolomucci A (2017). Stress, overeating, and obesity: Insights from human studies and preclinical models. Neurosci. Biobehav. Rev..

[3] Stenvinkel P (2015). Obesity—a disease with many aetiologies disguised in the same oversized phenotype: has the overeating theory failed?. Nephrol. Dial. Transplant..

[4] Bowe B (2018). The 2016 global and national burden of diabetes mellitus attributable to PM_2·5_ air pollution. Lancet Planet Health..

[5] An R, Ji M, Yan H, Guan C (2018). Impact of ambient air pollution on obesity: a systematic review. Int. J. Obes. (Lond)..

[6] Landrigan PJ (2018). The Lancet Commission on pollution and health. Lancet.

[7] Bell ML (2007). Climate change, ambient ozone, and health in 50 US cities. Clim. Change.

[8] Gackière F, Saliba L, Baude A, Bosler O, Strube C (2011). Ozone inhalation activates stress-responsive regions of the CNS. J. Neurochem..

[9] Nowotny B (2010). Effects of acute psychological stress on glucose metabolism and subclinical inflammation in patients with post-traumatic stress disorder. Horm. Metab. Res..

[10] Snow SJ, Henriquez AR, Costa DL, Kodavanti UP (2018). Neuroendocrine regulation of air pollution health effects: emerging insights. Toxicol. Sci..

[11] Kodavanti UP (2019). Susceptibility variations in air pollution health effects: Incorporating neuroendocrine activation. Toxicol. Pathol..

[12] Shankar K (2017). Environmental forces that shape early development: What we know and still need to know. Curr. Dev. Nutr..

[13] Lomas-Soria C (2018). Maternal obesity has sex-dependent effects on insulin, glucose and lipid metabolism and the liver transcriptome in young adult rat offspring. J. Physiol..

[14] Lewis RM, Desoye G (2017). Placental lipid and fatty acid transfer in maternal overnutrition. Ann. Nutr. Metab..

[15] Puppala S (2018). Primate fetal hepatic responses to maternal obesity: epigenetic signalling pathways and lipid accumulation. J. Physiol..

[16] Hjort L (2018). Gestational diabetes and maternal obesity are associated with epigenome-wide methylation changes in children. JCI Insight..

[17] Gomes RM (2018). Maternal diet-induced obesity during suckling `period programs offspring obese phenotype and hypothalamic leptin/insulin resistance. J. Nutr. Biochem..

[18] Inadera H (2013). Developmental origins of obesity and type 2 diabetes: molecular aspects and role of chemicals. Environ. Health. Prev. Med..

[19] Wankhade UD, Thakali KM, Shankar K (2016). Persistent influence of maternal obesity on offspring health: Mechanisms from animal models and clinical studies. Mol. Cell. Endocrinol..

[20] Green BB, Marsit CJ (2015). Select prenatal environmental exposures and subsequent alterations of gene-specific and repetitive element DNA methylation in fetal tissues. Curr. Environ. Health. Rep..

[21] Liu Y, Peterson KE (2015). Maternal exposure to synthetic chemicals and obesity in the offspring: Recent findings. Curr. Environ. Health. Rep..

[22] Bass V (2013). Ozone induces glucose intolerance and systemic metabolic effects in young and aged Brown Norway rats. Toxicol. Appl. Pharmacol..

[23] Miller DB (2015). Inhaled ozone (O3)-induces changes in serum metabolomic and liver transcriptomic profiles in rats. Toxicol. Appl. Pharmacol..

[24] Miller DB (2016). Acute ozone-induced pulmonary and systemic metabolic effects are diminished in adrenalectomized rats. Toxicol. Sci..

[25] Miller DB (2016). Systemic metabolic derangement, pulmonary effects, and insulin insufficiency following subchronic ozone exposure in rats. Toxicol. Appl. Pharmacol..

[26] Snow SJ (2017). Respiratory effects and systemic stress response following acute acrolein inhalation in rats. Toxicol. Sci..

[27] Miller DB (2016). Ozone exposure increases circulating stress hormones and lipid metabolites in humans. Am. J. Respir. Crit. Care Med..

[28] Kodavanti UP (2016). Stretching the stress boundary: Linking air pollution health effects to a neurohormonal stress response. Biochim. Biophys. Acta..

[29] Gordon CJ (2017). Effects of maternal high-fat diet and sedentary lifestyle on susceptibility of adult offspring to ozone exposure in rats. Inhal. Toxicol..

[30] Snow SJ (2019). Maternal high-fat diet alters offspring response to an acute ozone exposure. J. Toxicol. Environ. Health Part A.

[31] Kodavanti UP (2015). Variability in ozone-induced pulmonary injury and inflammation in healthy and cardiovascular-compromised rat models. Inhal. Toxicol..

[32] Grissom NM, George R, Reyes TM (2017). The hypothalamic transcriptional response to stress is severely impaired in offspring exposed to adverse nutrition during gestation. Neuroscience.

[33] Klein MO (2018). POMC and NPY mRNA expression during development is increased in rat offspring brain from mothers fed with a high fat diet. Int. J. Dev. Neurosci..

[34] Gostner JM (2019). Tryptophan metabolism and related pathways in psychoneuroimmunology: The impact of nutrition and lifestyle. Neuropsychobiology.

[35] Lent-Schochet D, McLaughlin M, Ramakrishnan N, Jialal I (2019). Exploratory metabolomics of metabolic syndrome: A status report. World J. Diabetes..

[36] Moser VC, Phillips PM, Hedge JM, McDaniel KL (2015). Neurotoxicological and thyroid evaluations of rats developmentally exposed to tris(1,3-dichloro-2-propyl)phosphate (TDCIPP) and tris(2-chloro-2-ethyl)phosphate (TCEP). Neurotoxicol. Teratol..

[37] Neuwirth LS, Phillips GR, El Idrissi A (2018). Perinatal Pb_2+_ exposure alters the expression of genes related to the neurodevelopmental GABA-shift in postnatal rats. J. Biomed. Sci..

[38] Moser VC (2017). Impacts of maternal diet and exercise on offspring behavior and body weights. Neurotoxicol. Teratol..

[39] Boukouvalas G, Gerozissis K, Markaki E, Kitraki E (2010). High-fat feeding influences the endocrine responses of pubertal rats to an acute stress. Neuroendocrinology.

[40] Mulligan CM, Friedman JE (2017). Maternal modifiers of the infant gut microbiota: metabolic consequences. J. Endocrinol..

[41] Guo Y (2018). Diet induced maternal obesity affects offspring gut microbiota and persists into young adulthood. Food Funct..

[42] Joëls M, Sarabdjitsingh RA, Karst H (2012). Unraveling the time domains of corticosteroid hormone influences on brain activity: rapid, slow, and chronic modes. Pharmacol. Rev..

[43] Kroetz DL, Xu F (2005). Regulation and inhibition of arachidonic acid omega-hydroxylases and 20-HETE formation. Annu. Rev. Pharmacol. Toxicol..

[44] Pietrocola F, Galluzzi L, Bravo-SanPedro JM, Madeo F, Kroemer G (2015). Acetyl coenzyme A: a central metabolite and second messenger. Cell Metab..

[45] Cristofano A (2016). Serum levels of acyl-carnitines along the continuum from normal to Alzheimer's dementia. PLoS One.

[46] Davies (2016). The acetyl group buffering action of carnitine acetyltransferase offsets macronutrient-induced lysine acetylation of mitochondrial proteins. Cell Rep..

[47] Hussain T (2017). Polyamines: therapeutic perspectives in oxidative stress and inflammatory diseases. Amino Acids.

[48] Hoet PH, Nemery B (2000). Polyamines in the lung: polyamine uptake and polyamine-linked pathological or toxicological conditions. Am. J. Physiol. Lung Cell Mol. Physiol..

[49] Elsayed NM, Ellingson AS, Tierney DF, Mustafa MG (1990). Effects of ozone inhalation on polyamine metabolism and tritiated thymidine incorporation into DNA of rat lungs. Toxicol. Appl. Pharmacol..

[50] Yeung AW, Terentis AC, King NJ, Thomas SR (2015). Role of indoleamine 2,3-dioxygenase in health and disease. Clin. Sci. (Lond).

[51] Badawy AA (2017). Tryptophan availability for kynurenine pathway metabolism across the life span: Control mechanisms and focus on aging, exercise, diet and nutritional supplements. Neuropharmacology.

[52] Van der Leek AP, Yanishevsky Y, Kozyrskyj AL (2017). The kynurenine pathway as a novel link between allergy and the gut microbiome. Front. Immunol..

[53] Pereira TJ (2015). Maternal obesity characterized by gestational diabetes increases the susceptibility of rat offspring to hepatic steatosis via a disrupted liver metabolome. J. Physiol..

[54] Chu DM, Meyer KM, Prince AL, Aagaard KM (2016). Impact of maternal nutrition in pregnancy and lactation on offspring gut microbial composition and function. Gut Microbes..

[55] Gubert C, Kong G, Renoir T, Hannan AJ (2020). Exercise, diet and stress as modulators of gut microbiota: Implications for neurodegenerative diseases. Neurobiol. Dis..

[56] Kriaa A (2019). Microbial impact on cholesterol and bile acid metabolism: current status and future prospects. J Lipid Res..

[57] Henriquez AR (2019). Ozone-induced dysregulation of neuroendocrine axes requires adrenal-derived stress hormones. Toxicol. Sci..

[58] Lutz TA, Wood SC (2012). Overview of animal models of obesity. Protoc. Pharmacol. Curr..

[59] Hatch GE (2013). Biomarkers of dose and effect of inhaled ozone in resting versus exercising human subjects: Comparison with resting rats. Biomark. Insights..

[60] Evans AM (2014). High resolution mass spectrometry improves data quantity and quality as compared to unit mass resolution mass spectrometry in high-throughput profiling metabolomics. Metabolomics.

