# Supplementary material for: Offspring susceptibility to metabolic alterations due to maternal high-fat diet and the impact of inhaled ozone used as a stressor
Source: Sci Rep. 2020 Oct 1;10:16353. doi: 10.1038/s41598-020-73361-0 (PMC7530537; doi:10.1038/s41598-020-73361-0)
Supplement: Supplementary file 1 — Supplementary Information 1 [file 41598_2020_73361_MOESM1_ESM.pdf]

On-line data supplement

Offspring Susceptibility to Metabolic Alterations Due to Maternal High-Fat Diet and The Impact of Inhaled Ozone Used as a Stressor

Samantha J. Snow[1*], Katarzyna Broniowska[2], Edward D. Karoly[2], Andres R. Henriquez[3], Pamela M. Phillips[1], Allen D. Ledbetter[1], Mette C. Schladweiler[1], Colette N. Miller[3], Christopher J. Gordon[1], Urmila P. Kodavanti[1**]

[1]Public Health and Integrated Toxicology Division, Center for Public Health and Environmental Assessment, US Environmental Protection Agency, Durham, NC, USA

[2]Metabolon Inc., Durham, NC, USA

[3]Oak Ridge Institute for Science and Education, Durham, NC, USA

[†]Current address: ICF, Durham, NC, USA

Running Title: Ozone-induced effects modified by maternal diet

Corresponding Author:

[1]Urmila P. Kodavanti, PhD

Public Health and Integrated Toxicology Division

Center for Public Health and Environmental Assessment

U.S. Environmental Protection Agency

109 T.W. Alexander Dr.

Research Triangle Park, NC 27711 USA

Tel.: +1 919 541 4963

Fax: +1 919 541 0026

E-mail: kodavanti.urmila@epa.gov

Supplementary Methods and Materials:

Supplementary Table 1. Control diet and high-fat diet composition.

Major Ingredients	Control Diet	High-Fat Diet
Casein, g/kg	210	265
L-Cystine, g/kg	3	4
Corn starch, g/kg	465	--
Maltodextrin, g/kg	100	160
Sucrose, g/kg	90	90
Lard, g/kg	20	310
Soybean oil, g/kg	20	30
Cellulose, g/kg	37	65.5

CD: %Kcal from: protein – 20.5, carbohydrate - 69.1, fat – 10.4

HFD: %Kcal from: protein – 18.4, carbohydrate – 21.3, fat – 60.3

Control diet (CD; ~10% of calories from fat; TD.08806) and high-fat diet (HFD; ~60% of calories from fat; TD.06414) from Harlan Laboratories (Teklad Custom Research Diets; Madison, WI) were shipped to Charles River Inc, Raleigh NC and to CPHEA, US EPA, Research Triangle Park, NC Animal Facility (Table 1).

Supplementary Figure Legends

Supplementary Figure 1. Bronchoalveolar lavage fluid (BALF) protein leakage, injury markers and neutrophilia after a single ozone exposure in offspring from dams fed a control diet (CD) or high-fat diet (HFD). Lungs were lavaged within 2h after a single 5h air or 0.8 ppm ozone exposure, and BALF levels for markers of injury (albumin, total protein, NAG activity) and inflammation (neutrophils) were analyzed. Values represent mean ± SEM, n=10/group. *Indicate significantly different from air group within same diet ($p<0.05$). P40, post-natal day 40; NAG, N-acetylglucosaminidase.

Supplementary Figure 2. Serum neuroendocrine hormone levels after a single ozone exposure in offspring from control diet (CD) or high-fat diet (HFD) dams. Serum samples were collected 2h after a single 5h air or 0.8 ppm ozone exposure. Values represent mean ± SEM, n=10/group. *Indicate significantly different from air group within same diet ($p<0.05$). † Indicate significantly different from control diet group within same exposure ($p<0.05$). P40, post-natal day 40; ACTH, adrenocorticotropic hormone; BDNF, brain-derived neurotrophic factor; FSH, follicle stimulating hormone; GH, growth hormone; PRL, prolactin; LH, luteinizing hormone; TSH, thyroid stimulating hormone.

Supplementary Figure 3. Circulating corticosterone and adrenaline after a single ozone exposure in offspring from control diet (CD) or high-fat diet (HFD) dams. Serum samples were collected 2h after a single 5h air or 0.8 ppm ozone exposure. Values represent mean ± SEM, n=10/group. P40, post-natal day 40.

Supplementary Figure 4. Serum insulin and leptin after a single ozone exposure in offspring from control diet (CD) or high-fat diet (HFD) dams. Serum samples were collected 2h after a single 5h air or 0.8 ppm ozone exposure. Values represent mean ± SEM, n=10/group. P40, post-natal day 40.

Supplementary Figure 5. Serum lipids and cholesterols after a single ozone exposure in offspring from control diet (CD) or high-fat diet (HFD) dams. Serum samples were collected 2h after a single 5h air or 0.8 ppm ozone exposure. Values represent mean ± SEM, n=10/group. † Indicate significantly different from control diet group within same exposure ($p<0.05$). P40, post-natal day 40; HDL, high-density lipoprotein, LDL, low-density lipoprotein, FFA, free fatty acids.

Supplementary Figure 6. A heat map showing metabolites that were significantly changed by maternal high-fat diet (HFD) in male and female offspring exposed to filtered air. The values show mean fold change due to HFD when compared to control diet (CD) (n=8/group). Red or green indicates $p\leq0.05$; pink or light green indicates $0.05<p<0.10$.

Supplementary Figure 7A-E. Heat maps showing significant ozone-induced changes in circulating lipid metabolites of male and female offspring from control diet (CD) ot high-fat diet (HFD) fed dams. The values show mean fold change due to ozone exposure when compared to filtered air in offspring (n=8/group). Red or green indicates $p\leq0.05$; pink or light green indicates $0.05<p<0.10$.

Supplementary Materials, Figure 8. Heat map showing ozone-induced significant changes in circulating carbohydrate metabolites of male and female offspring from control diet (CD) or high fat diet (HFD) fed dams. The values show mean fold change due to ozone exposure when compared to filtered air in offspring (n=8/group). Red or green indicates $p \leq 0.05$; pink or light green indicates $0.05 < p < 0.10$.

Supplementary Materials, Figure 9A-B. Heat maps showing significant ozone-induced changes in circulating amino acid metabolites of male and female offspring from control diet (CD) or high fat diet (HFD) fed dams. The values show mean fold change due to ozone exposure when compared to filtered air in offspring (n=8/group). Red or green indicates $p \leq 0.05$; pink or light green indicates $0.05 < p < 0.10$.

Supplementary Figure 1

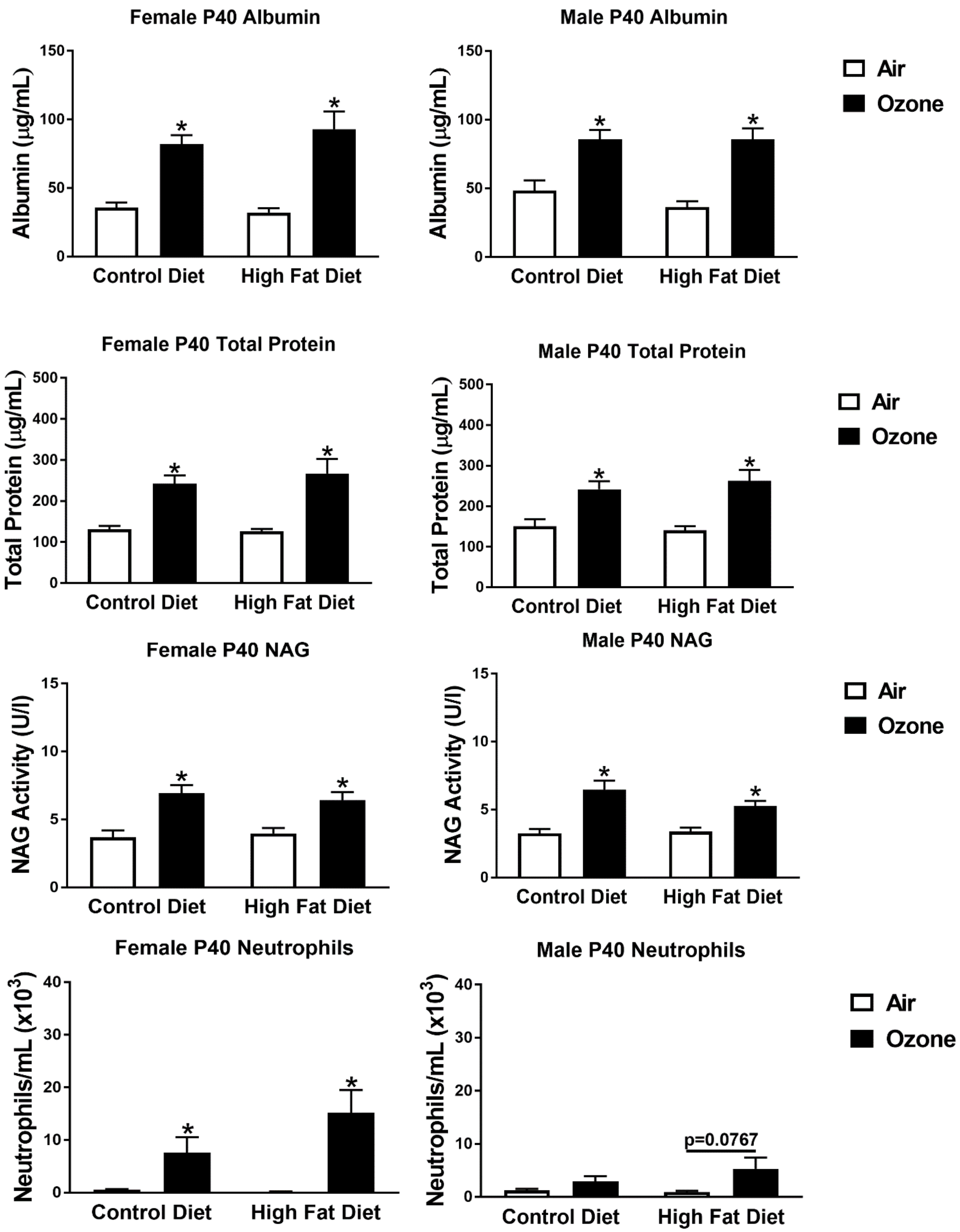

Supplementary Figure 2

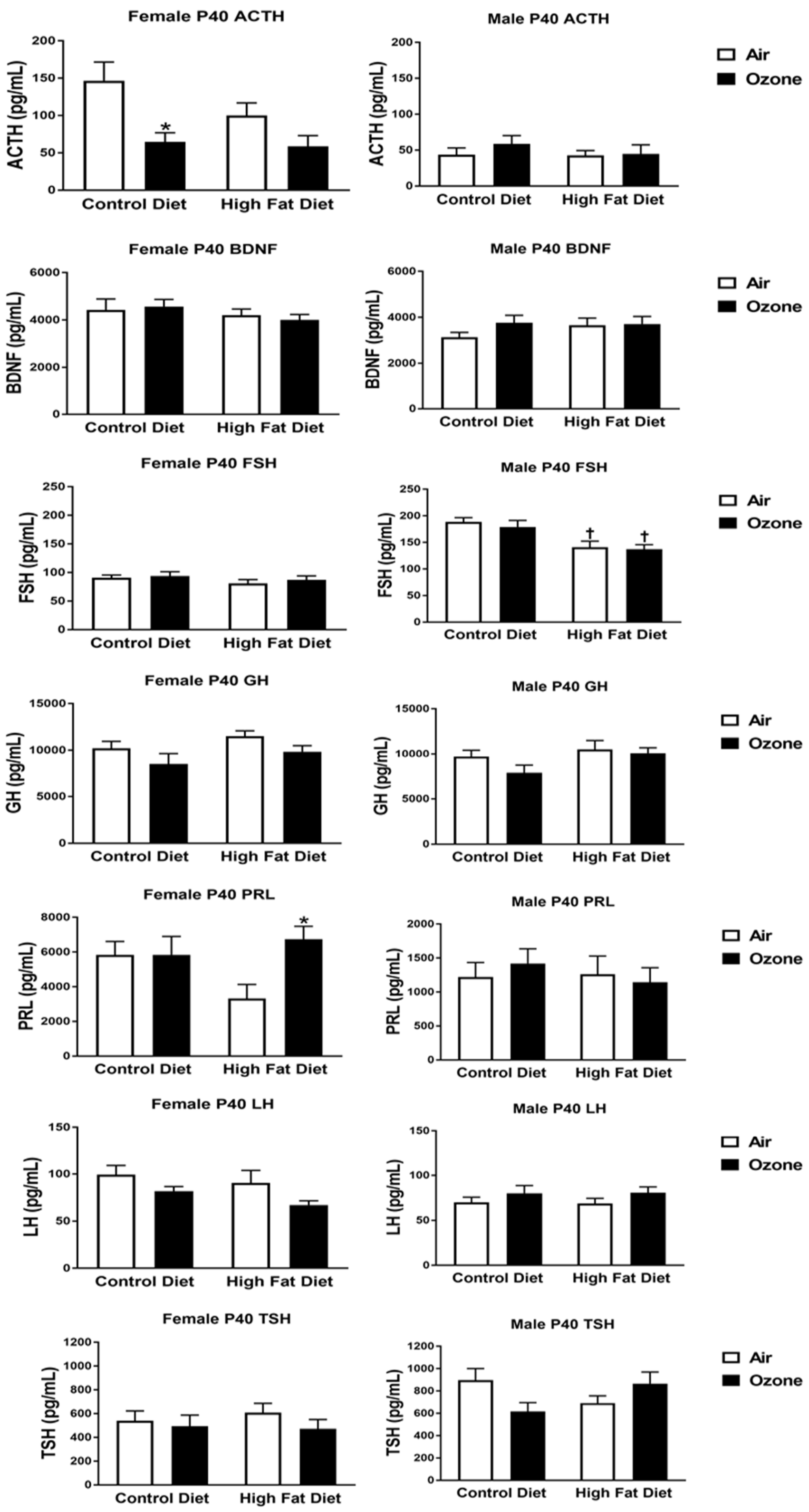

Supplementary Figure 3

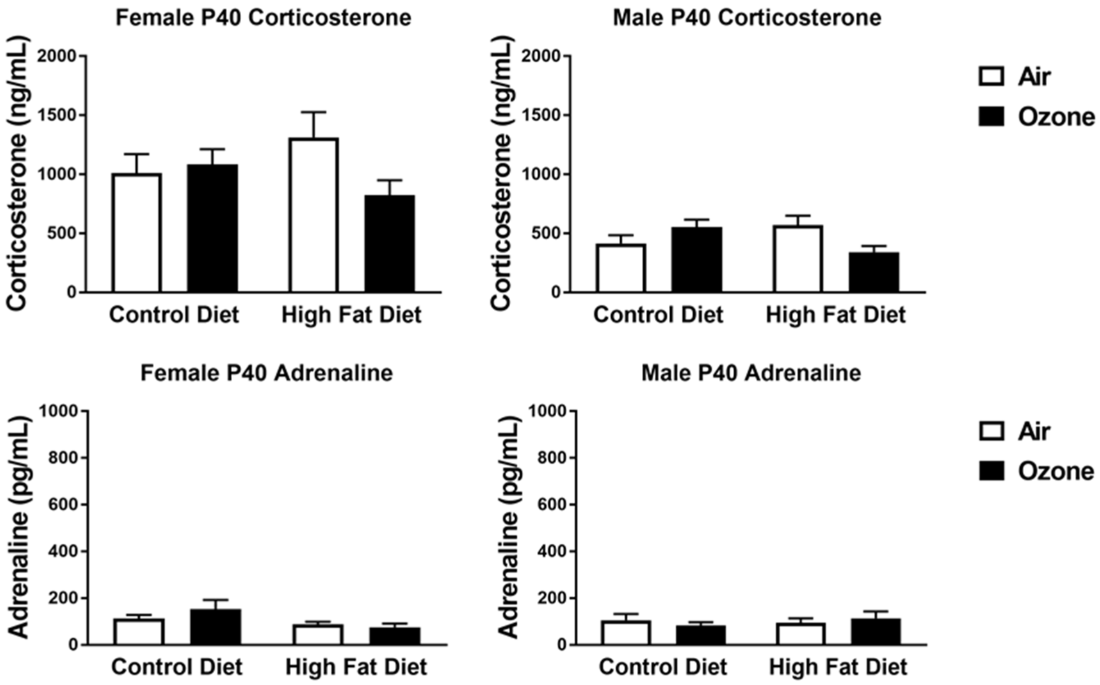

Supplementary Figure 4

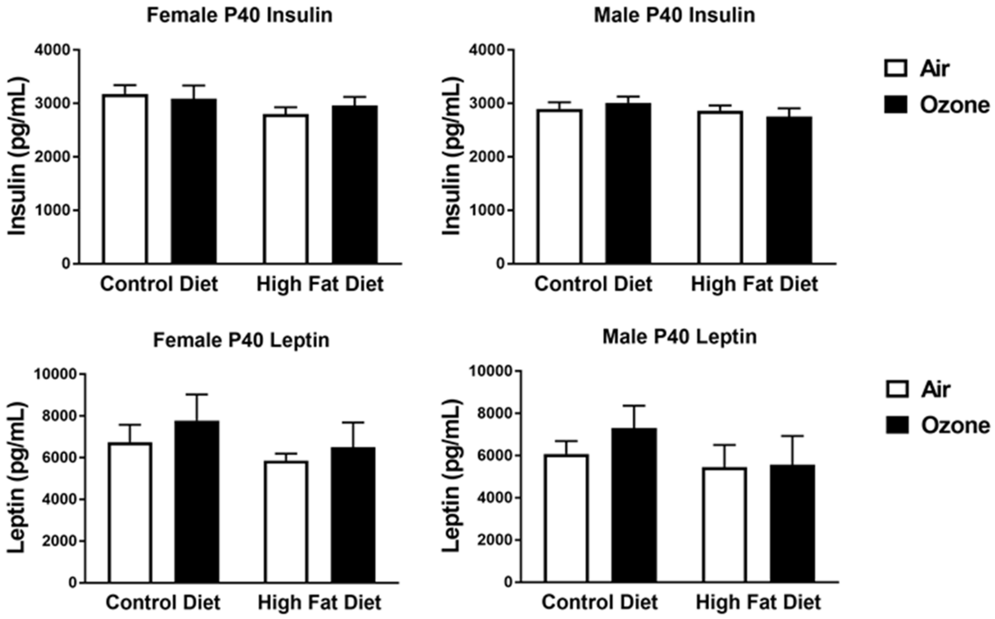

Supplementary Figure 5

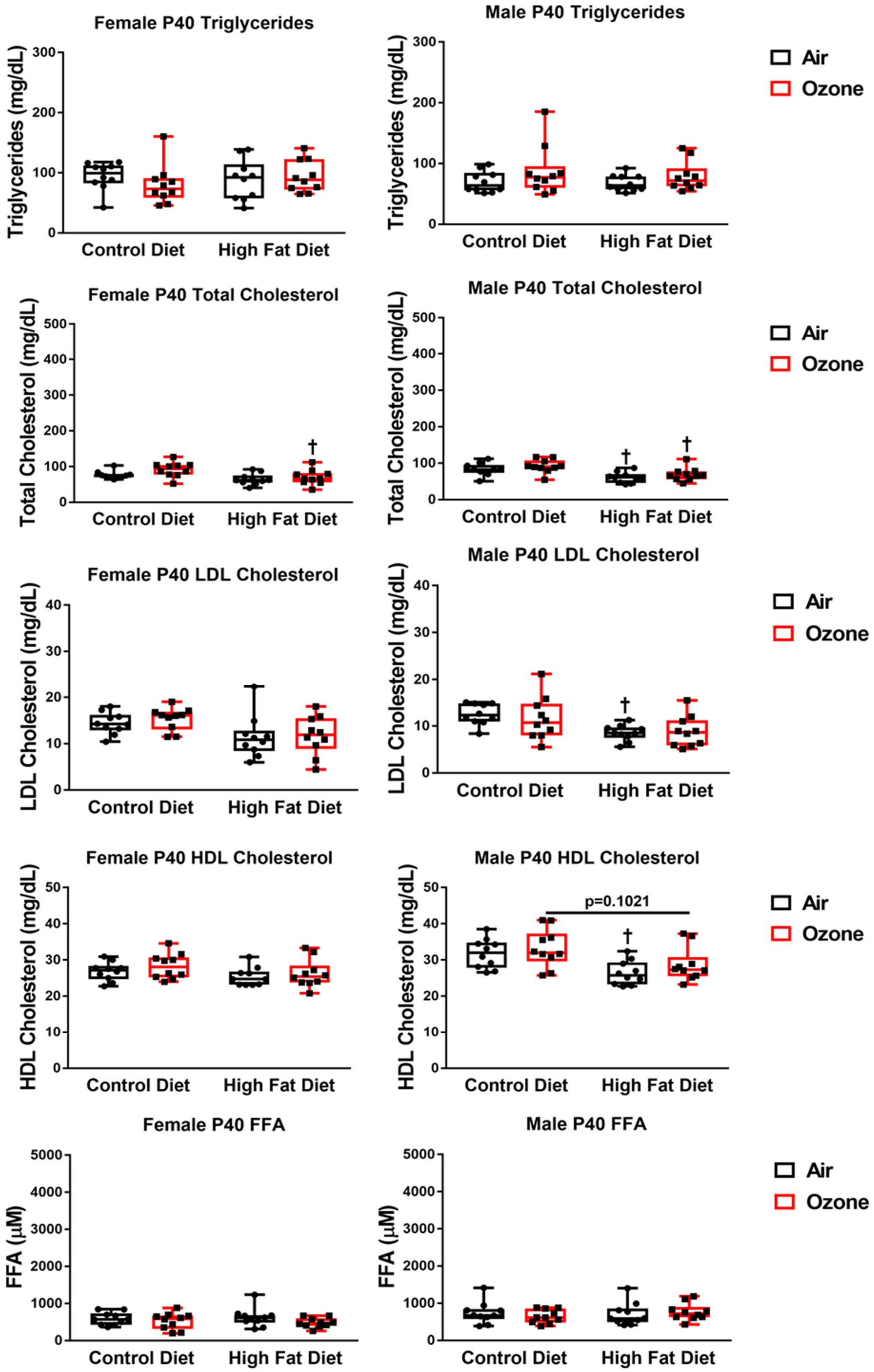

Supplementary Figure 6

Sub pathway	Biochemical Name	Female HFD:Air/CD:Air	Male HFD:Air/CD:Air
Tyrosine Metabolism	phenol sulfate	3.07	0.82
	phenol glucuronide	2.90	0.85
	vanillactate	0.70	1.30
	p-cresol-glucuronide*	0.41	0.52
	thyroxine	1.18	1.32
	caproate (6:0)	1.30	1.16
Medium Chain Fatty Acid	caprylate (8:0)	0.99	1.23
	caprate (10:0)	0.60	0.53
	10-undecenoate (11:1n1)	0.71	0.77
	laurate (12:0)	0.50	0.47
	myristate (14:0)	0.90	0.72
Long Chain Fatty Acid	margarate (17:0)	1.21	1.40
	10-heptadecenoate (17:1n7)	1.35	1.25
	stearate (18:0)	1.08	1.24
	nonadecanoate (19:0)	1.01	1.25
	10-nonadecenoate (19:1n9)	1.29	1.48
Polyunsaturated Fatty Acid (n3 and n6)	heneicosapentaenoate (21:5n3)	1.57	1.06
	hexadecadienoate (16:2n6)	1.45	1.69
	docosapentaenoate (n3 DPA; 22:5n3)	1.32	1.47
	linoleate (18:2n6)	1.39	1.49
	arachidonate (20:4n6)	1.03	1.19
	adrenate (22:4n6)	1.45	1.37
	docosadienoate (22:2n6)	1.05	1.31
Fatty Acid Metabolism (also BCAA Metabolism)	dihomo-linoleate (20:2n6)	1.61	1.84
	propionylcarnitine (C3)	0.99	0.77
	propionylglycine	0.94	1.43
	methylmalonate (MMA)	1.05	0.80
	3-hydroxybutyrylcarnitine (1)	0.77	0.81
	3-hydroxybutyrylcarnitine (2)	0.80	1.11
	hexanoylcarnitine (C6)	0.92	0.79
	decanoylcarnitine (C10)	1.04	0.74
	laurylcarnitine (C12)	0.86	0.68
	stearoylcarnitine (C18)	1.21	1.22
	linoleoylcarnitine (C18:2)*	1.32	1.48
	arachidoylcarnitine (C20)*	1.18	1.01
	dihomo-linoleoylcarnitine (C20:2)*	1.85	2.35
	margaroylcarnitine (C17)*	1.15	1.56
	1-palmitoyl-2-arachidonoyl-GPE (16:0/20:4)*	1.01	1.35
	1-stearoyl-2-arachidonoyl-GPE (18:0/20:4)	1.09	1.40
	1-stearoyl-2-docosahexaenoyl-GPE (18:0/22:6)*	0.98	1.43
Phosphatidylinositol (PI)	1-palmitoyl-2-oleoyl-GPI (16:0/18:1)*	0.73	0.89
	1-palmitoyl-2-linoleoyl-GPI (16:0/18:2)	0.68	0.96
	1-palmitoyl-2-arachidonoyl-GPI (16:0/20:4)*	0.77	1.08

Sub pathway	Biochemical Name	Female HFD:Air/CD:Air	Male HFD:Air/CD:Air
Glucose	1,5-anhydroglucitol (1,5-AG)	0.92	0.88
	pyruvate	1.06	0.79
Lysophospholipid	1-linoleoyl-GPA (18:2)*	0.84	0.74
	1-palmitoleoyl-GPC (16:1)*	0.98	0.80
	2-palmitoleoyl-GPC (16:1)*	0.82	0.68
	1-linoleoyl-GPC (18:2)	0.99	0.86
	1-palmitoyl-GPE (16:0)	1.01	1.11
	1-stearoyl-GPE (18:0)	1.04	1.11
	2-stearoyl-GPE (18:0)*	0.97	1.19
	1-oleoyl-GPG (18:1)*	1.08	0.72
Monoacylglycerol	1-myristoylglycerol (14:0)	0.95	0.83
	1-palmitoleoylglycerol (16:1)*	0.83	0.78
	1-oleoylglycerol (18:1)	0.64	0.63
	1-linoleoylglycerol (18:2)	0.87	0.84
	1-dihomo-linolenylglycerol (20:3)	0.76	0.46
	2-oleoylglycerol (18:1)	0.84	0.64
Diacylglycerol	diacylglycerol (14:0/18:1, 16:0/16:1) [2]*	0.88	0.61
	palmitoleoyl-palmitoleoyl-glycerol (16:1/16:1) [2]*	1.01	0.50
	palmitoyl-oleoyl-glycerol (16:0/18:1) [2]*	0.94	0.69
	oleoyl-arachidonoyl-glycerol (18:1/20:4) [1]*	1.19	1.43
	oleoyl-arachidonoyl-glycerol (18:1/20:4) [2]*	1.25	1.49
	linoleoyl-arachidonoyl-glycerol (18:2/20:4) [1]*	1.18	1.78
	linoleoyl-arachidonoyl-glycerol (18:2/20:4) [2]*	1.23	1.93
Ceramides	N-stearoyl-sphingosine (d18:1/18:0)*	1.40	1.07
	ceramide (d18:1/17:0, d17:1/18:0)*	1.47	1.13
	ceramide (d18:2/24:1, d18:1/24:2)*	1.20	1.02
	glycosyl-N-stearoyl-sphingosine (d18:1/18:0)	1.58	0.88
	glycosyl-N-arachidoyl-sphingosine (d18:1/20:0)*	1.62	0.88
	glycosyl ceramide (d18:1/23:1, d17:1/24:1)*	1.62	0.81
	glycosyl ceramide (d18:2/24:1, d18:1/24:2)*	1.50	0.96
Sterol	7-alpha-hydroxy-3-oxo-4-cholestenoate (7-Hoca)	0.57	0.76
	3beta,7alpha-dihydroxy-5-cholestenoate	0.47	0.73
	beta-sitosterol	0.78	0.82
	campesterol	0.83	0.89
Pyrimidine Metabolism, Cytidine containing	cytidine	1.25	1.39
	5-methylcytidine	1.00	1.11
	2'-deoxycytidine	1.04	1.13
Benzoate Metabolism	hippurate	0.66	0.80
	catechol sulfate	0.60	0.56
	4-methylcatechol sulfate	0.58	0.67
	4-ethylphenylsulfate	0.45	0.39
	p-cresol sulfate	0.47	0.57
	3-(3-hydroxyphenyl)propionate sulfate	1.11	2.06

Supplementary Figure 7A

Sub Pathway	Biochemical Name	Exposure Effect			
		F:CD:O3/ F:CD:Air	F:HFD:O3/ F:HFD:Air	M:CD:O3/ M:CD:Air	M:HFD:O3/ M:HFD:Air
Short Chain Fatty Acid	valerate (5:0)	0.30	0.39	0.42	0.45
Medium Chain Fatty Acid	caproate (6:0)	0.55	0.41	0.77	0.56
	caprylate (8:0)	0.48	0.39	0.79	0.54
	caprate (10:0)	0.70	0.74	0.73	0.84
	10-undecenoate (11:1n1)	0.69	0.73	0.81	1.05
	laurate (12:0)	0.60	0.77	0.72	0.84
	5-dodecenoate (12:1n7)	0.47	0.43	0.70	0.67
Long Chain Fatty Acid	myristate (14:0)	0.93	0.82	0.90	1.04
	myristoleate (14:1n5)	0.76	0.56	0.90	0.92
	pentadecanoate (15:0)	1.04	1.04	0.98	1.06
	palmitate (16:0)	0.97	0.79	1.02	1.01
	palmitoleate (16:1n7)	0.97	0.69	1.08	1.06
	margarate (17:0)	1.00	0.86	0.96	0.99
	10-heptadecenoate (17:1n7)	1.05	0.72	0.97	1.02
	stearate (18:0)	1.12	0.96	1.13	1.06
	oleate/vaccenate (18:1)	0.99	0.73	1.10	1.01
	nonadecanoate (19:0)	1.09	1.02	0.99	0.91
	10-nonadecenoate (19:1n9)	0.96	0.79	1.11	0.99
	arachidate (20:0)	1.24	1.33	1.08	1.10
	eicosenoate (20:1)	1.14	0.92	1.19	0.91
	erucate (22:1n9)	1.00	1.05	0.91	1.09
Polyunsaturated Fatty Acid (n3 and n6)	heneicosapentaenoate (21:5n3)	1.73	1.01	1.55	1.55
	hexadecadienoate (16:2n6)	0.85	0.68	0.92	1.00
	stearidonate (18:4n3)	0.96	0.97	1.04	1.03
	eicosapentaenoate (EPA; 20:5n3)	1.02	0.74	1.09	1.08
	docosapentaenoate (n3 DPA; 22:5n3)	1.29	1.03	1.33	1.17
	docosahexaenoate (DHA; 22:6n3)	1.24	0.96	1.35	1.20
	docosatrienoate (22:3n3)	1.25	1.04	1.53	1.12
	nisinate (24:6n3)	1.31	0.93	1.90	1.24
	linoleate (18:2n6)	0.80	0.64	0.89	0.96
	linolenate [alpha or gamma; (18:3n3 or 6)]	0.77	0.60	0.84	0.96
	dihomo-linolenate (20:3n3 or n6)	1.10	0.84	1.33	1.10
	arachidonate (20:4n6)	1.02	0.94	1.22	1.01
	adrenate (22:4n6)	1.29	0.90	1.43	1.30
	docosapentaenoate (n6 DPA; 22:5n6)	1.33	0.89	1.56	1.08
	docosadienoate (22:2n6)	1.08	1.09	1.19	1.00
	dihomo-linoleate (20:2n6)	1.08	0.82	1.28	1.01
	mead acid (20:3n9)	1.21	0.82	1.48	1.01
	docosatrienoate (22:3n6)*	1.35	1.11	1.72	1.13

Supplementary Figure 7B

Sub Pathway	Biochemical Name	Exposure Effect			
		F:CD:O3/ F:CD:Air	F:HFD:O3/ F:HFD:Air	M:CD:O3/ M:CD:Air	M:HFD:O3/ M:HFD:Air
Fatty Acid, Dicarboxylate	dimethylmalonic acid	0.93	0.83	0.63	1.39
	glutarate (C5-DC)	1.51	1.29	0.96	1.04
	2-hydroxyglutarate	0.95	1.06	1.34	0.91
	2-hydroxyadipate	2.32	2.47	1.94	1.46
	3-hydroxyadipate*	0.84	0.82	0.82	0.85
	pimelate (C7-DC)	2.87	1.98	1.94	3.10
	heptenedioate (C7:1-DC)*	1.05	1.18	0.81	1.12
	suberate (C8-DC)	1.04	0.77	0.84	1.30
	azelate (C9-DC)	1.61	1.40	1.52	1.48
	sebacate (C10-DC)	0.53	0.59	0.68	1.24
	undecanedioate (C11-DC)	1.72	1.27	1.52	2.46
	dodecanedioate (C12-DC)	0.59	0.71	0.71	1.10
	tetradecanedioate (C14-DC)	0.73	0.65	0.74	1.09
	hexadecanedioate (C16-DC)	0.60	0.44	0.77	1.16
	hexadecenedioate (C16:1-DC)*	0.63	0.45	0.90	1.18
	octadecanedioate (C18-DC)	0.68	0.46	0.93	1.63
	octadecenedioate (C18:1-DC)*	0.58	0.49	0.95	1.28
	eicosanodioate (C20-DC)	0.84	0.53	0.93	1.03
	docosadioate (C22-DC)	0.85	0.48	0.72	0.80
Fatty Acid Metabolism (also BCAA Metabolism)	butyrylcarnitine (C4)	1.25	1.37	1.33	0.97
	butyrylglycine	0.58	0.74	0.64	1.00
	propionylcarnitine (C3)	1.34	1.23	1.38	1.30
	propionylglycine	1.36	1.53	1.38	1.09
	methylmalonate (MMA)	1.50	1.34	1.29	1.34
Fatty Acid Metabolism (Acyl Carnitine)	acetylcarnitine (C2)	0.80	0.80	1.03	1.08
	3-hydroxybutyrylcarnitine (1)	1.02	1.27	0.97	1.02
	3-hydroxybutyrylcarnitine (2)	0.77	1.17	1.00	0.93
	hexanoylcarnitine (C6)	0.72	0.74	0.87	1.16
	octanoylcarnitine (C8)	0.77	0.66	0.85	1.10
	decanoylcarnitine (C10)	0.70	0.61	0.74	0.95
	5-dodecenoylcarnitine (C12:1)	0.68	0.63	0.75	0.95
	cis-4-decenoylcarnitine (C10:1)	0.77	0.63	0.77	0.91
	laurylcarnitine (C12)	0.67	0.72	0.73	0.93
	myristoylcarnitine (C14)	0.66	0.68	0.69	0.81
	palmitoylcarnitine (C16)	0.81	0.75	0.80	0.83
	palmitoleoylcarnitine (C16:1)*	0.59	0.58	0.69	0.88
	stearoylcarnitine (C18)	1.03	0.84	0.97	0.90
	linoleoylcarnitine (C18:2)*	0.67	0.60	0.69	0.87
	linolenoylcarnitine (C18:3)*	0.55	0.49	0.56	0.86
	oleoylcarnitine (C18:1)	0.77	0.67	0.82	0.92
	myristoleoylcarnitine (C14:1)*	0.65	0.58	0.77	0.91
	octadecanedioylcarnitine (C18-DC)*	0.86	1.04	1.30	1.15
	octadecenedioylcarnitine (C18:1-DC)*	0.88	0.79	0.63	0.95
	arachidoylcarnitine (C20)*	1.04	0.87	1.01	0.90
	arachidonoylcarnitine (C20:4)	0.82	0.67	1.03	1.22
	behenoylcarnitine (C22)*	1.04	1.09	1.29	1.09
	dihomo-linolenoylcarnitine (20:3n3 or 6)*	0.64	0.54	0.90	1.06
	dihomo-linoleoylcarnitine (C20:2)*	0.83	0.52	1.32	1.04
	eicosenoylcarnitine (C20:1)*	0.87	0.62	0.99	0.99
	docosahexaenoylcarnitine (C22:6)*	0.68	0.83	0.73	1.33
	lignoceroylcarnitine (C24)*	1.05	0.90	0.99	0.95
	margaroylcarnitine (C17)*	0.89	0.81	0.80	0.90
	ximenoylcarnitine (C26:1)*	0.86	0.89	0.82	0.74
	pentadecanoylcarnitine (C15)*	0.76	0.68	0.67	0.79

Supplementary Figure 7C

Sub Pathway	Biochemical Name	Exposure Effect			
		F:CD:O3/ F:CD:Air	F:HFD:O3/ F:HFD:Air	M:CD:O3/ M:CD:Air	M:HFD:O3/ M:HFD:Air
Ketone Bodies	3-hydroxybutyrate (BHBA)	0.54	0.64	0.59	0.72
Fatty Acid Metabolism (Acyl Choline)	palmitoylcholine	2.66	1.79	2.04	1.34
	oleoylcholine	2.88	1.53	2.42	1.42
	palmitoloelycholine	2.83	1.51	2.24	1.09
	dihomo-linolenoyl-choline	4.70	1.84	3.12	1.76
	linoleoylcholine*	2.80	1.63	1.72	1.30
	stearoylcholine*	2.98	1.58	2.39	1.65
	docosahexaenoylcholine	2.58	1.54	2.06	1.49
	arachidonoylcholine	2.80	1.55	2.25	1.48
Fatty Acid, Monohydroxy	2-hydroxyoctanoate	1.05	0.67	1.09	1.61
	2-hydroxydecanoate	0.88	0.67	0.91	0.98
	2-hydroxymyristate	1.31	0.87	0.98	1.42
	2-hydroxyoleate	0.96	0.77	1.12	1.28
	2-hydroxypalmitate	1.05	0.90	1.08	1.07
	2-hydroxystearate	1.15	1.01	1.18	1.13
	2-hydroxybehenate	0.92	0.91	0.98	1.07
	3-hydroxyhexanoate	0.62	0.83	0.71	1.29
	3-hydroxyoctanoate	0.45	0.76	0.64	1.39
	3-hydroxydecanoate	0.29	0.32	0.56	0.56
	3-hydroxyoleate*	0.89	0.68	1.08	0.96
	3-hydroxysebacate	0.54	0.53	0.70	1.23
	3-hydroxylaurate	0.54	0.55	0.80	1.12
	3-hydroxymyristate	0.76	0.74	0.88	1.00
	5-hydroxyhexanoate	0.64	0.82	0.88	0.82
	16-hydroxypalmitate	0.81	0.52	1.01	0.87
	13-HODE + 9-HODE	0.80	0.66	0.97	0.82
	14-HDoHE/17-HDoHE	1.03	1.24	1.25	1.15
	3-hydroxystearate	1.26	0.90	1.53	1.23
Fatty Acid, Dihydroxy	12,13-DiHOME	0.98	0.93	1.20	1.03
	9,10-DiHOME	0.80	0.60	0.86	1.19
Eicosanoid	thromboxane B2	0.75	1.34	1.32	0.57
	12-HEPE	0.95	1.12	1.34	1.24
	12-HETE	0.98	1.39	1.34	1.01
	12-HHTrE	0.98	1.35	1.21	0.69
Endocannabinoid	oleoyl ethanolamide	1.08	0.96	1.04	1.09
	palmitoyl ethanolamide	1.03	1.01	1.01	0.95
	N-oleoyltaurine	1.02	0.93	1.04	1.14
	N-stearoyltaurine	1.10	1.11	1.12	1.09
	N-palmitoyltaurine	1.02	0.97	1.07	1.29
	N-linoleoyltaurine*	0.88	0.98	0.96	1.18
	linoleoyl ethanolamide	0.94	0.79	1.05	1.07
Inositol Metabolism	myo-inositol	1.07	1.38	1.21	1.34

Supplementary Figure 7D

Sub Pathway	Biochemical Name	Exposure Effect			
		F:CD:O3/ F:CD:Air	F:HFD:O3/ F:HFD:Air	M:CD:O3/ M:CD:Air	M:HFD:O3/ M:HFD:Air
Phosphatidylcholine (PC)	1-myristoyl-2-palmitoyl-GPC (14:0/16:0)	0.83	1.17	0.85	0.85
	1-myristoyl-2-arachidonoyl-GPC (14:0/20:4)*	0.89	1.16	1.01	0.89
	1,2-dipalmitoyl-GPC (16:0/16:0)	0.93	1.02	0.95	0.89
	1-palmitoyl-2-palmitoleoyl-GPC (16:0/16:1)*	0.86	1.00	0.98	0.87
	1-palmitoyl-2-stearoyl-GPC (16:0/18:0)	0.83	0.86	0.89	0.81
	1-palmitoyl-2-oleoyl-GPC (16:0/18:1)	0.92	0.94	0.94	0.88
	1-palmitoyl-2-linoleoyl-GPC (16:0/18:2)	0.95	1.07	0.84	0.87
	1-palmitoyl-2-gamma-linolenoyl-GPC (16:0/18:3n6)*	0.82	0.99	0.81	0.86
	1,2-distearoyl-GPC (18:0/18:0)	0.79	0.71	0.94	0.87
	1-stearoyl-2-oleoyl-GPC (18:0/18:1)	1.01	0.96	1.07	1.02
	1-stearoyl-2-linoleoyl-GPC (18:0/18:2)*	1.01	1.06	0.97	0.91
	1-stearoyl-2-arachidonoyl-GPC (18:0/20:4)	1.04	1.00	1.08	1.03
	1-stearoyl-2-docosahexaenoyl-GPC (18:0/22:6)	1.04	1.02	1.09	1.05
	1,2-dilinoleoyl-GPC (18:2/18:2)	0.97	1.24	0.69	0.81
Phosphatidylethanolamine (PE)	1-palmitoyl-2-oleoyl-GPE (16:0/18:1)	1.09	1.34	0.84	1.05
	1-palmitoyl-2-linoleoyl-GPE (16:0/18:2)	0.96	1.61	0.62	0.96
	1-stearoyl-2-linoleoyl-GPE (18:0/18:2)*	1.04	1.39	0.77	1.08
	1-stearoyl-2-arachidonoyl-GPE (18:0/20:4)	1.00	1.19	0.87	0.99
	1-stearoyl-2-docosahexaenoyl-GPE (18:0/22:6)*	0.94	1.16	0.84	0.91
	1-oleoyl-2-linoleoyl-GPE (18:1/18:2)*	0.91	1.72	0.70	1.01
Phosphatidylinositol (PI)	1-palmitoyl-2-oleoyl-GPI (16:0/18:1)*	1.01	1.30	0.66	0.78
	1-palmitoyl-2-linoleoyl-GPI (16:0/18:2)	0.86	1.61	0.76	0.81
	1-palmitoyl-2-arachidonoyl-GPI (16:0/20:4)*	0.84	1.29	0.89	0.88
	1-stearoyl-2-linoleoyl-GPI (18:0/18:2)	0.98	0.98	0.72	1.04
	1-stearoyl-2-arachidonoyl-GPI (18:0/20:4)	0.99	1.04	1.00	0.94
Lysophospholipid	1-palmitoyl-GPA (16:0)	1.11	1.59	1.10	0.96
	1-palmitoleoyl-GPA (16:1)*	0.85	1.28	1.28	0.91
	1-stearoyl-GPA (18:0)	1.43	1.42	1.21	0.77
	1-oleoyl-GPA (18:1)	1.06	1.29	1.17	1.09
	1-linoleoyl-GPA (18:2)*	1.02	1.36	0.97	1.14
	1-arachidonoyl-GPA (20:4)	0.86	0.95	1.05	0.98
	1-palmitoyl-GPC (16:0)	0.97	0.93	0.94	0.83
	1-oleoyl-GPC (18:1)	0.91	0.81	0.95	0.88
	1-linoleoyl-GPC (18:2)	0.81	0.85	0.78	0.81
	1-linolenoyl-GPC (18:3)*	0.72	0.71	0.65	0.66
	1-arachidonoyl-GPC (20:4n6)*	0.90	0.83	0.96	0.96
	1-lignoceroyl-GPC (24:0)	1.10	1.12	0.98	0.97
	1-palmitoyl-GPE (16:0)	1.00	1.04	0.91	0.86
	1-stearoyl-GPE (18:0)	0.96	0.95	0.86	0.83
	2-stearoyl-GPE (18:0)*	0.96	1.07	1.04	0.86
	1-oleoyl-GPE (18:1)	0.99	0.98	0.96	0.99
	1-linoleoyl-GPE (18:2)*	1.02	1.18	0.76	0.97
	1-oleoyl-GPS (18:1)	0.94	1.61	1.23	1.36
	1-linoleoyl-GPS (18:2)*	1.18	1.75	1.25	1.49
	1-palmitoyl-GPG (16:0)*	1.35	1.23	1.09	1.22
	1-oleoyl-GPG (18:1)*	1.38	0.95	1.11	1.38
	1-linoleoyl-GPG (18:2)*	1.30	1.08	1.10	1.50
	1-palmitoyl-GPI (16:0)	1.40	1.48	1.48	1.24
	1-stearoyl-GPI (18:0)	1.61	1.48	1.58	1.19
	1-oleoyl-GPI (18:1)*	1.59	1.45	1.43	1.29
	1-linoleoyl-GPI (18:2)*	1.96	1.61	1.31	1.64
	1-arachidonoyl-GPI (20:4)*	1.53	1.28	1.50	1.26

Supplementary Figure 7E

Sub Pathway	Biochemical Name	Exposure Effect			
		F:CD:O3/ F:CD:Air	F:HFD:O3/ F:HFD:Air	M:CD:O3/ M:CD:Air	M:HFD:O3/ M:HFD:Air
Monoacylglycerol	1-myristoylglycerol (14:0)	1.29	1.36	1.30	1.17
	1-palmitoylglycerol (16:0)	1.24	1.12	1.10	1.11
	1-palmitoleoylglycerol (16:1)*	1.21	1.18	1.46	1.09
	1-oleoylglycerol (18:1)	1.08	1.39	1.41	1.52
	1-arachidonylglycerol (20:4)	1.26	1.12	1.29	1.23
	1-docosahexaenoylglycerol (22:6)	1.41	1.31	1.46	1.47
	2-palmitoylglycerol (16:0)	1.68	0.97	1.31	1.28
	2-palmitoleoylglycerol (16:1)*	1.20	1.09	1.69	1.02
	2-oleoylglycerol (18:1)	1.14	0.96	1.26	1.40
	2-linoleoylglycerol (18:2)	1.06	1.10	1.03	1.14
	2-arachidonoylglycerol (20:4)	1.33	1.16	1.37	1.22
Diacylglycerol	diacylglycerol (16:1/18:2 [2], 16:0/18:3 [1])*	0.65	0.82	0.92	1.10
	palmitoleoyl-linoleoyl-glycerol (16:1/18:2) [1]*	0.55	0.80	0.89	1.07
	oleoyl-linoleoyl-glycerol (18:1/18:2) [1]	0.69	0.89	0.93	1.06
	oleoyl-linoleoyl-glycerol (18:1/18:2) [2]	0.66	0.90	0.93	0.95
	oleoyl-linolenoyl-glycerol (18:1/18:3) [2]*	0.40	0.59	0.69	0.82
	linoleoyl-linolenoyl-glycerol (18:2/18:3) [2]*	0.47	0.80	0.68	0.80
	stearoyl-arachidonoyl-glycerol (18:0/20:4) [1]*	1.27	1.17	1.31	1.15
	stearoyl-arachidonoyl-glycerol (18:0/20:4) [2]*	1.32	1.14	1.18	0.98
	oleoyl-arachidonoyl-glycerol (18:1/20:4) [2]*	1.05	0.99	1.32	1.22
Sphingolipid Metabolism	sphinganine	0.84	1.28	1.07	1.36
	N-arachidoyl-sphingosine (d18:1/20:0)*	1.04	0.75	1.01	0.96
	N-palmitoyl-sphinganine (d18:0/16:0)	0.85	0.86	0.94	0.92
	N-behenoyl-sphingadienine (d18:2/22:0)*	0.87	0.80	0.94	0.76
	palmitoyl sphingomyelin (d18:1/16:0)	1.10	1.00	1.02	1.05
	stearoyl sphingomyelin (d18:1/18:0)	1.24	1.02	1.09	0.98
	behenoyl sphingomyelin (d18:1/22:0)*	1.10	1.06	1.01	0.96
	tricosanoyl sphingomyelin (d18:1/23:0)*	1.16	1.10	1.01	1.05
	lignoceroyl sphingomyelin (d18:1/24:0)	1.20	1.08	1.02	1.00
	sphingomyelin (d18:2/16:0, d18:1/16:1)*	1.13	1.05	1.06	1.02
	sphingomyelin (d18:1/18:1, d18:2/18:0)	1.12	1.02	1.08	1.01
	sphingomyelin (d18:1/20:0, d16:1/22:0)*	1.18	1.09	1.05	0.96
	sphingomyelin (d18:1/20:1, d18:2/20:0)*	1.18	1.13	1.21	1.06
	sphingomyelin (d18:1/22:1, d18:2/22:0, d16:1/24:1)*	1.14	1.09	1.07	0.95
	sphingomyelin (d18:1/24:1, d18:2/24:0)*	1.12	0.99	1.05	1.00
	sphingomyelin (d18:2/24:1, d18:1/24:2)*	1.11	1.11	1.02	0.97
	sphingosine 1-phosphate	1.10	1.07	1.18	1.12
	phytosphingosine	1.04	1.18	1.15	1.31
	sphingomyelin (d18:2/23:1)*	1.03	1.23	0.92	0.98
	sphingomyelin (d18:1/20:2, d18:2/20:1, d16:1/22:2)*	1.25	1.21	1.45	1.32
	sphingomyelin (d18:2/24:2)*	1.14	1.16	0.92	1.04
	sphingomyelin (d18:0/20:0, d16:0/22:0)*	1.35	1.06	0.82	1.12
	sphingomyelin (d18:0/18:0, d19:0/17:0)*	1.19	1.09	0.96	1.06
	sphingomyelin (d17:2/16:0, d18:2/15:0)*	1.17	1.27	0.98	1.00
	sphingomyelin (d18:2/18:1)*	1.19	1.18	1.25	1.15
Sterol	cholesterol	1.16	0.99	1.19	1.10
	7-alpha-hydroxy-3-oxo-4-cholestenoate (7-Hoca)	2.21	2.21	1.69	1.71
	3beta,7alpha-dihydroxy-5-cholestenoate	3.01	3.06	1.60	1.41
	3beta-hydroxy-5-cholestenoate	0.96	0.72	1.06	0.95
	4-cholesten-3-one	1.01	0.63	1.00	0.90
	7-hydroxycholesterol (alpha or beta)	5.57	4.92	1.86	1.59

Supplementary Figure 8

Sub Pathway	Biochemical Name	Exposure Effect			
		F:CD:O3/ F:CD:Air	F:HFD:O3/ F:HFD:Air	M:CD:O3/ M:CD:Air	M:HFD:O3/ M:HFD:Air
Glycolysis, Gluconeogenesis, and Pyruvate Metabolism	1,5-anhydroglucitol (1,5-AG)	1.01	0.96	1.12	1.00
	glucose	1.07	1.06	**1.13**	**1.18**
	pyruvate	**1.41**	**1.60**	**1.24**	1.23
	lactate	0.99	**1.30**	1.29	**1.40**
Pentose metabolism	ribitol	1.18	**1.49**	1.10	**1.26**
	xylose	1.22	**1.54**	1.13	1.20
	arabinose	**1.56**	**2.29**	**1.88**	2.36
	arabitol/xylitol	1.13	**1.23**	1.02	**1.24**
	arabonate/xylonate	**1.22**	1.01	1.03	1.13
	ribulonate/xylulonate*	1.07	1.12	**1.23**	**1.23**
Fructose, Mannose and Galactose Metabolism	fructose	1.18	1.21	1.19	**1.19**
	mannitol/sorbitol	1.14	**1.25**	1.15	1.21
	mannose	**0.81**	**0.72**	0.89	0.95
Aminosugar Metabolism	glucuronate	1.12	1.19	1.10	**1.25**
	N-acetylneuraminate	**1.24**	1.20	1.07	1.21
	erythronate*	0.99	1.08	**0.86**	1.01
	N-acetylglucosamine/N-acetylgalactosamine	**0.86**	0.90	0.97	1.03
TCA Cycle	citrate	1.03	**1.29**	1.01	0.97
	aconitate [cis or trans]	0.98	**1.17**	0.95	**0.88**
	isocitrate	1.02	**1.29**	1.05	0.90
	succinylcarnitine (C4-DC)	**1.98**	**2.33**	1.01	1.12
	malate	**0.85**	1.13	1.00	1.11
	2-methylcitrate/homocitrate	**0.76**	**0.78**	1.06	**0.57**
Oxidative Phosphorylation	phosphate	1.02	1.00	**0.95**	**0.94**

Supplementary Figure 9A

Sub Pathway	Biochemical Name	Exposure Effect			
		F:CD:O3/F:CD:Air	F:HFD:O3/F:HFD:Air	M:CD:O3/M:CD:Air	M:HFD:O3/M:HFD:Air
Glycine metabolism	sarcosine	1.86	1.88	0.96	0.86
	dimethylglycine	1.34	1.40	1.00	1.10
Glutamate metabolism	alpha-ketoglutaramate**	1.24	1.17	1.10	1.03
	N-acetylglutamate	1.49	1.60	1.27	1.26
	4-hydroxyglutamate	0.98	1.82	1.05	1.54
	N-acetyl-aspartyl-glutamate (NAAG)	1.01	1.53	1.01	1.14
Histidine Metabolism	histidine	1.13	1.12	1.13	1.10
	1-methylhistidine	0.81	1.03	0.94	0.72
	3-methylhistidine	0.90	1.06	0.94	0.70
	N-acetylhistidine	1.79	1.64	1.59	1.22
	trans-urocanate	1.81	0.92	1.91	1.04
	formiminoglutamate	1.92	0.50	0.75	0.48
	imidazole lactate	1.20	1.02	1.45	0.78
	carnosine	1.13	1.89	1.40	1.07
	N-acetylcarnosine	0.96	1.45	1.23	0.69
	anserine	1.13	1.78	1.43	1.07
	N-acetylhistamine	1.60	2.29	1.26	1.51
Lysine metabolism	N6,N6,N6-trimethyllysine	1.11	1.26	1.07	1.08
	5-(galactosylhydroxy)-L-lysine	0.93	1.45	1.13	1.15
	2-oxoadipate	1.49	1.46	1.34	1.06
	6-oxopiperidine-2-carboxylate	1.07	0.93	0.84	0.78
	5-aminovalerate	1.77	1.15	0.84	0.95
Phenylalanine Metabolism	phenylalanine	1.08	1.09	1.18	1.18
	N-acetylphenylalanine	2.45	2.29	1.71	1.57
	phenylpyruvate	0.34	0.51	0.58	0.26
	phenyllactate (PLA)	5.22	2.76	1.34	1.74
	phenylacetate	1.24	1.21	1.22	2.18
Tyrosine Metabolism	tyrosine	0.83	0.92	0.99	0.95
	N-acetyltyrosine	1.76	1.48	1.08	1.07
	4-hydroxyphenylpyruvate	0.77	0.88	0.72	0.46
	3-(4-hydroxyphenyl)lactate	1.13	1.32	0.99	0.91
	vanillactate	1.80	2.46	1.25	0.72
	3-methoxytyrosine	0.92	0.89	0.86	0.77
	thyroxine	0.74	0.82	1.03	0.78
Tryptophan Metabolism	tryptophan	0.99	0.93	1.05	0.96
	N-acetyltryptophan	1.33	1.42	0.98	0.82
	C-glycosyltryptophan	0.96	1.03	0.90	1.09
	kynurenine	1.33	1.39	1.02	1.04
	N-acetylkynurenine (2)	1.26	1.44	1.02	1.03
	kynurenate	1.56	1.77	1.10	1.45
	anthranilate	1.19	1.45	1.27	1.40
	xanthurenate	1.80	2.33	1.17	1.61
	picolinate	1.37	1.32	1.27	0.97
	serotonin	1.13	1.17	1.30	1.16
	5-hydroxyindoleacetate	1.06	1.45	0.98	1.12
Leucine, Isoleucine and Valine Metabolism	N-acetylleucine	2.23	1.53	1.74	1.47
	alpha-hydroxyisocaproate	1.58	1.33	1.20	1.35
	isovalerylglycine	1.52	2.04	1.53	1.53
	isovalerylcarnitine (C5)	1.43	1.47	1.79	1.80
	beta-hydroxyisovalerate	0.95	1.06	0.92	1.33
	3-methylglutaconate	1.33	1.45	0.98	1.19
	N-acetylisoleucine	2.62	1.90	2.06	1.30
	3-methyl-2-oxovalerate	0.99	1.18	1.13	1.11
	alpha-hydroxyisovalerate	1.03	1.30	1.05	1.20
	3-hydroxy-2-ethylpropionate	1.08	1.24	0.96	0.84
	ethylmalonate	1.55	1.63	1.09	1.15
	valine	1.04	0.99	1.17	1.04
	N-acetylvaline	1.53	1.35	1.44	1.10
	3-methyl-2-oxobutyrate	1.02	1.06	1.19	1.13
	2-hydroxy-3-methylvalerate	1.55	1.60	1.11	1.25
	isobutyrylcarnitine (C4)	1.26	1.37	1.46	1.66
	isobutyrylglycine	0.97	1.19	1.03	1.82
	3-hydroxyisobutyrate	1.53	1.73	1.12	0.83

Supplementary Figure 9B

Sub Pathway	Biochemical Name	Exposure Effect			
		F:CD:O3/F:CD:Air	F:HFD:O3/F:HFD:Air	M:CD:O3/M:CD:Air	M:HFD:O3/M:HFD:Air
Methionine, Cysteine, SAM and Taurine Metabolism	methionine	0.99	1.07	1.09	1.06
	N-acetylmethionine	0.92	1.35	1.12	1.13
	N-formylmethionine	0.98	1.25	1.15	1.01
	methionine sulfone	0.95	1.37	1.22	1.25
	methionine sulfoxide	1.22	1.43	1.16	1.19
	N-acetylmethionine sulfoxide	1.12	1.07	1.29	2.87
	5-methylthioribose**	0.71	0.94	0.81	0.76
	S-adenosylhomocysteine (SAH)	0.98	1.51	1.01	0.79
	cystathionine	1.11	1.33	1.17	0.75
	alpha-ketobutyrate	1.18	1.06	1.61	1.04
	cysteine	1.05	1.29	1.23	0.78
	S-methylcysteine	1.08	0.93	1.18	1.01
	cysteine s-sulfate	0.76	1.33	0.96	1.92
	hypotaurine	1.17	1.20	1.40	1.28
	taurine	0.98	1.13	1.07	1.08
	N-acetyltaurine	1.23	1.31	1.09	0.99
Urea cycle; Arginine and Proline Metabolism	argininosuccinate	1.05	1.51	0.72	0.94
	urea	1.23	1.18	0.95	1.22
	ornithine	0.92	0.61	1.11	1.05
	homoarginine	0.99	1.44	0.96	0.89
	homocitrulline	0.77	0.88	1.03	0.86
	proline	1.06	1.34	1.14	1.07
	dimethylarginine (SDMA + ADMA)	0.91	1.03	0.97	1.00
	N-acetylarginine	0.98	1.27	1.24	0.86
	N-acetylcitrulline	1.37	1.54	1.06	1.12
	N-acetylproline	1.08	1.41	1.14	0.93
	trans-4-hydroxyproline	0.80	0.91	0.82	0.85
Polyamine Metabolism	putrescine	1.45	1.38	1.26	1.24
	spermidine	1.46	1.46	1.41	1.34
	5-methylthioadenosine (MTA)	1.00	1.46	1.03	1.10
	N-acetylputrescine	1.10	1.49	1.16	1.16
	4-acetamidobutanoate	1.09	1.34	1.08	1.04
	(N(1) + N(8))-acetylspermidine	1.45	1.47	1.23	1.30

Supplementary Materials, Results:

Supplementary Materials, Figures 1. Maternal high fat diet (HFD) was not associated with significant changes in any of the BALF indices of lung injury and inflammation (air-exposed rats). However, as expected a single 5-hr 0.8 ppm ozone exposure increased in BALF protein and albumin in both male and female offspring indicating vascular leakage. N-acetylglucosaminidase (activity), a lysosomal enzyme indicative of macrophage activation was increased in BALF after ozone exposure in both male and female offspring from dams fed control diet (CD) and HFD. Increases in BALF neutrophils was readily noted at this timepoint, indicating an activation of an innate immune response after ozone exposure. Females appeared to be more affected by ozone. Male and female offspring from HFD group appeared to have a slightly more increases than respective CD groups.

Supplementary Materials, Figure 2: A panel of pituitary hormones were assessed in the serum since we have shown that ozone exposure is associated with the activation of neuroendocrine axes. At the time point, the effect was examined, ozone decreased ACTH in female offspring from CD dams whereas increased prolactin in offspring from HFD dams. No other significant ozone-related changes were observed in male or female offspring from either diet. Dam HFD in male offspring resulted in decreased levels of FSH.

Supplementary Materials, Figure 3. Since short ozone exposure has been associated with increases in corticosterone and epinephrine in male WKY rats (Miller et al., 2016), we determined these hormones in male and female offspring. No ozone, or diet-related changes were apparent in this strain and with current study design, however, in general males tended to have low levels of corticosterone when compared to female offspring. No effects of ozone, diet or sex on adrenaline (epinephrine) levels were noted. The levels of corticosterone and epinephrine after ozone exposure were variable. However, in other studies with 2 or 4 hours of ozone exposures, increases have been noted (Miller et al., 2016).

Supplementary Materials, Figure 4. There were no ozone or maternal diet-related changes in circulating insulin in 40-day old male or female offspring. Surprisingly leptin levels tended to be lower in both male and female offspring from HFD fed dams.

Supplementary Materials, Figure 5. Although ozone exposure did not change any of the lipid metabolites in the serum, maternal diet-related small changes were observed. Males and females offspring from HFD had slightly lower circulating cholesterol when compared to CD. Levels of LDL and HDL were lower in male offspring from HFD dams. No diet or exposure-related differences were observed in circulating free fatty acids. Since no gross significant sex differences were noted in any of the clinical measures of lipid metabolites in 40-day old rats although metabolomic analysis of specific lipid species indicated significant sex differences (discussed in the main manuscript).

Supplementary Materials, Figure 6: Maternal HFD-induced changes in circulating metabolites in male and female offspring exposed to filtered air are shown in the heat map. In general, maternal diet-related changes in tyrosine metabolism were noted in both male and female offspring. Medium chain fatty acids were largely decreased in both female and male offspring while long chain and poly unsaturated fatty acids were increased due to maternal HFD in both female and male offspring (male>female). Major changes in acyl carnitines were noted in males and females (males>females) while phosphatidyl inositol metabolites were decreased in females from HFD dams. Male offspring from HFD dams had decreased 1,5-anhydroglucitol (1,5-AG). Lysophospholipids and monoacylglycerols were decreased with increases in some diacylglycerols in males from HFD dams but no changes in females were noted. The levels of ceramides were increased only in the female rats from HFD dams. In general, few sterol metabolites were

decreased in both male and female offspring. And changes in the benzoate metabolites in both male and female offspring may indicate changes in gut microbiota.

Supplementary Materials Figure 7 A-E): Short-chain fatty acid, valerate (5:0) was decreased in all ozone-exposed offspring (CD>HFD). Likewise, medium chain fatty acids were decreased in female and male offspring from CD and HFD fed dams (Females>males), whereas long chain fatty acids were decreased only in females from HFD dams. Poly unsaturated fatty acids were increased after ozone exposure in males from CD dams but decreased in females from HFD dams (Supplementary Materials, Figure 7A). Short chain dicarboxylates were increased in all ozone-exposed offspring regardless of sex or maternal diet, however long-chain dicarboxylates were decreased only in female offspring (HFD>CD). Increases in metabolites related to branched-chain amino acid metabolism were noted in all ozone-exposed offspring. A large number of acyl carnitines were markedly decreased in female offspring from CD and HFD dams (HFD>CD) and only in males from CD dams (Supplementary Materials, Figure 7B). Supplementary Materials, Figure 7C shows that ketone bodies, 3-hydroxybutyrate (BHBA) was decreased after ozone exposure in male and female offspring from CD dams. A variety of acyl cholines were markedly increased in male and female offspring, from CD and HFD dams, but this effect was more robust in offspring from CD dams. Monohydroxy fatty acids were decreased after ozone exposure but primarily in female offspring from dams on both dietary groups. A number of phosphatidyl cholines were decreased in all offspring exposed to ozone. Phosphatidyl ethanolamines and inositols were increased after ozone exposure in female offspring from HFD dams but decreased in male offspring from CD dams. Ozone-induced decreases in glycerophosphocholine (GPC) (males and females) and glycerophosphoethanolamine (GPE) (males) and increases in glycerophosphoglycerol (GPG) and glycerophosphoinositol (GPI) lysophospholipids in all male and female offspring were likely indicative to changes in membrane phospholipid turnover (Supplementary Materials, Figure 7D). Ozone exposure was also associated with increases in monoacylglycerols in both female offspring from CD dams and male offspring from both CD and HFD dams. This increase was associated with decreases in diacyl glycerols but only in female offspring from CD dams. Ozone exposure related increases were noted in a variety of sphingolipid metabolites in male and female offspring from CD dams (females>males). Marked increases in sterol metabolites including 7-alpha-hydroxy-3-oxo-4-cholestenoate (7-Hoca) and 7-hydroxycholesterol especially in female offspring may indicate a link to gut microbiome changes (Supplementary Materials Figure 7E).

Supplementary Materials, Figure 8: Ozone -induced changes in carbohydrate metabolites in male and female offspring from CD and HFD dams are shown in the heat map. While glucose levels were increased in male offspring, the levels of pyruvate and lactate were increased in both female and male offspring from CD and HFD dams. Pentose pathway metabolites were primarily increased in ozone-exposed female offspring from HFD dams. This was in addition to increases in TCA cycle intermediates, such as citrate, aconitate, isocitrate and succinyl carnitine.

Supplementary Materials, Figure 9A-B: A variety of amino acid metabolites were markedly increased in ozone-exposed female offspring from CD and HFD dams (HFD>CD). These effects were relatively small in male offspring. The increases in amino acid metabolites included those involved in glycine, glutamate, histidine, lysine, tyrosine, tryptophan, leucine, isoleucine, valine methionine, cysteine, taurine, arginine, and polyamine metabolites.

References:

Miller DB, Snow SJ, Schladweiler MC, Richards JE, Ghio AJ, Ledbetter AD, Kodavanti UP. Acute Ozone-Induced Pulmonary and Systemic Metabolic Effects Are Diminished in Adrenalectomized Rats. Toxicol Sci. 2016 Apr;150(2):312-22.
